# Therapeutic targeting of CNBP phase separation inhibits ribosome biogenesis and neuroblastoma progression via modulating SWI/SNF complex activity

**DOI:** 10.1002/ctm2.1235

**Published:** 2023-04-26

**Authors:** Anpei Hu, Guo Chen, Banghe Bao, Yanhua Guo, Dan Li, Xiaojing Wang, Jianqun Wang, Qilan Li, Yi Zhou, Haiyang Gao, Jiyu Song, Xinyi Du, Liduan Zheng, Qiangsong Tong

**Affiliations:** ^1^ Department of Pediatric Surgery Union Hospital, Tongji Medical College, Huazhong University of Science and Technology Wuhan Hubei Province P. R. China; ^2^ Department of Pathology Union Hospital, Tongji Medical College, Huazhong University of Science and Technology Wuhan Hubei Province P. R. China; ^3^ Clinical Center of Human Genomic Research Union Hospital, Tongji Medical College, Huazhong University of Science and Technology Wuhan Hubei Province P. R. China; ^4^ Department of Gastrointestinal Surgery Union Hospital, Tongji Medical College, Huazhong University of Science and Technology Wuhan Hubei Province P. R. China

**Keywords:** cellular nucleic acid‐binding protein, karyopherin subunit beta 1, phase separation, ribosome biogenesis, SWI/SNF complex, tumour progression

## Abstract

**Background:**

Neuroblastoma (NB) is the most common extracranial malignancy in childhood; however, the mechanisms underlying its aggressive characteristics still remain elusive.

**Methods:**

Integrative data analysis was performed to reveal tumour‐driving transcriptional regulators. Co‐immunoprecipitation and mass spectrometry assays were applied for protein interaction studies. Real‐time reverse transcription‐polymerase chain reaction, western blotting, sequential chromatin immunoprecipitation and dual‐luciferase reporter assays were carried out to explore gene expression regulation. The biological characteristics of NB cell lines were examined via gain‐ and loss‐of‐function assays. For survival analysis, the Cox regression model and log‐rank tests were used.

**Results:**

Cellular nucleic acid‐binding protein (*CNBP*) was found to be an independent factor affecting NB outcome, which exerted oncogenic roles in ribosome biogenesis, tumourigenesis and aggressiveness. Mechanistically, karyopherin subunit beta 1 (KPNB1) was responsible for nuclear transport of CNBP, whereas liquid condensates of CNBP repressed the activity of switch/sucrose‐nonfermentable (SWI/SNF) core subunits (SMARCC2/SMARCC1/SMARCA4) via interaction with SMARCC2, leading to alternatively increased activity of SMARCC1/SMARCA4 binary complex in facilitating gene expression essential for 18S ribosomal RNA (rRNA) processing in tumour cells, extracellular vesicle‐mediated delivery of 18S rRNA and subsequent M2 macrophage polarisation. A cell‐penetrating peptide blocking phase separation and interaction of CNBP with SMARCC2 inhibited ribosome biogenesis and NB progression. High *KPNB1*, *CNBP*, *SMARCC1* or *SMARCA4* expression or low *SMARCC2* levels were associated with poor survival of NB patients.

**Conclusions:**

These findings suggest that CNBP phase separation is a target for inhibiting ribosome biogenesis and tumour progression in NB via modulating SWI/SNF complex activity.

## Graphical abstract

1. CNBP is associated with poor prognosis and clinical progression of neuroblastoma.

2. KPNB1 facilitates nuclear translocation of CNBP to interact with SMARCC2 in liquid condensates.

3. CNBP modulates SWI/SNF activity via SMARCC2 to drive ribosome biogenesis of neuroblastoma cells and subsequent M2 macrophage polarisation.

4. Therapeutic targeting of phase separation and interaction of CNBP with SMARCC2 inhibits neuroblastoma progression.

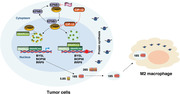

## 1 BACKGROUND

As the most common extracranial malignancy arising from neural crest, neuroblastoma (NB) occurs mainly in adrenal medulla or sympathetic ganglia during childhood^1^ and is characterised by striking heterogeneity of clinical and biological features.^1^ Currently, NB cases are stratified according to age at diagnosis, histopathological presentation, *MYCN* amplification, disease stage, DNA ploidy or chromosomal aberrations.^1^, ^2^ Despite rigorous chemotherapy, radiotherapy or surgical treatment, the prognosis of NB patients in high‐risk group is still dismal, with long‐term survival rates fewer than 40%.^1^ Thus, it is urgent to identify targets driving tumourigenesis and aggressiveness of NB, which is important for risk stratification and accurate therapeutics of patients.

Cellular nucleic acid‐binding protein (CNBP) is a conserved protein of 19 kDa,^3^ while amplification of its CCTG repeat within intron 1 leads to myotonic dystrophy type 2, an autosomal dominant multisystem disease.^4^ As a nucleic acid chaperone, *CNBP* participates in negative or positive regulation of gene transcription, such as Wnt 5^5^ and interleukin 6.^6^ In gastric cancer, *CNBP* facilitates the growth and aggressive behaviours of cancer cells via up‐regulating human antigen R at the transcriptional levels.^7^ Additionally, *CNBP* facilitates the progression of melanoma and lung cancer by directly activating promoters of matrix metallopeptidase 2, matrix metallopeptidase 14 or E2F transcription factor 2.^8^ Meanwhile, through translational up‐regulation of ornithine decarboxylase, a regulator of polyamine synthesis, *CNBP* promotes the growth of medulloblastoma.^9^ However, the action modes and regulatory mechanisms of *CNBP* during NB progression are yet unknown.

In the current study, *CNBP* is discovered as an independent factor affecting NB outcome. *CNBP* exerts oncogenic roles in ribosome biogenesis, tumourigenesis and aggressiveness. Karyopherin subunit beta 1 (KPNB1) is responsible for nuclear transport of CNBP. By forming liquid condensates together with switch/sucrose‐nonfermentable (SWI/SNF)‐related matrix‐associated actin‐dependent regulator of chromatin (SMARC) subfamily c member 2 (SMARCC2), CNBP represses the activity of SMARCC2/SMARC subfamily c member 1 (SMARCC1)/SMARC subfamily a member 4 (SMARCA4), core subunits of mammalian SWI/SNF chromatin‐remodelling complex, leading to alternatively increased activity of SMARCC1/SMARCA4 binary complex in facilitating gene expression essential for 18S ribosomal RNA (rRNA) processing in tumour cells, which further activates M2 macrophage polarization via extracellular vesicle (EV)‐mediated 18S rRNA delivery. Pharmacological or genetic inhibition of *KPNB1*/*CNBP* and cell‐penetrating peptide blocking phase separation and interaction of CNBP with SMARCC2 are efficient in reducing tumourigenesis and aggressiveness. Clinical NB cases with up‐regulation of *KPNB1*, *CNBP*, *SMARCC1*, *SMARCA4* or down‐regulation of *SMARCC2* have poor survival and prognosis, indicating *KPNB1*/*CNBP*/*SMARCC2* axis as a valuable target for therapeutics of tumours.

## 2 RESULTS

### 2.1 *CNBP* is linked to poor prognosis and clinical progression of NB

**FIGURE 1:**
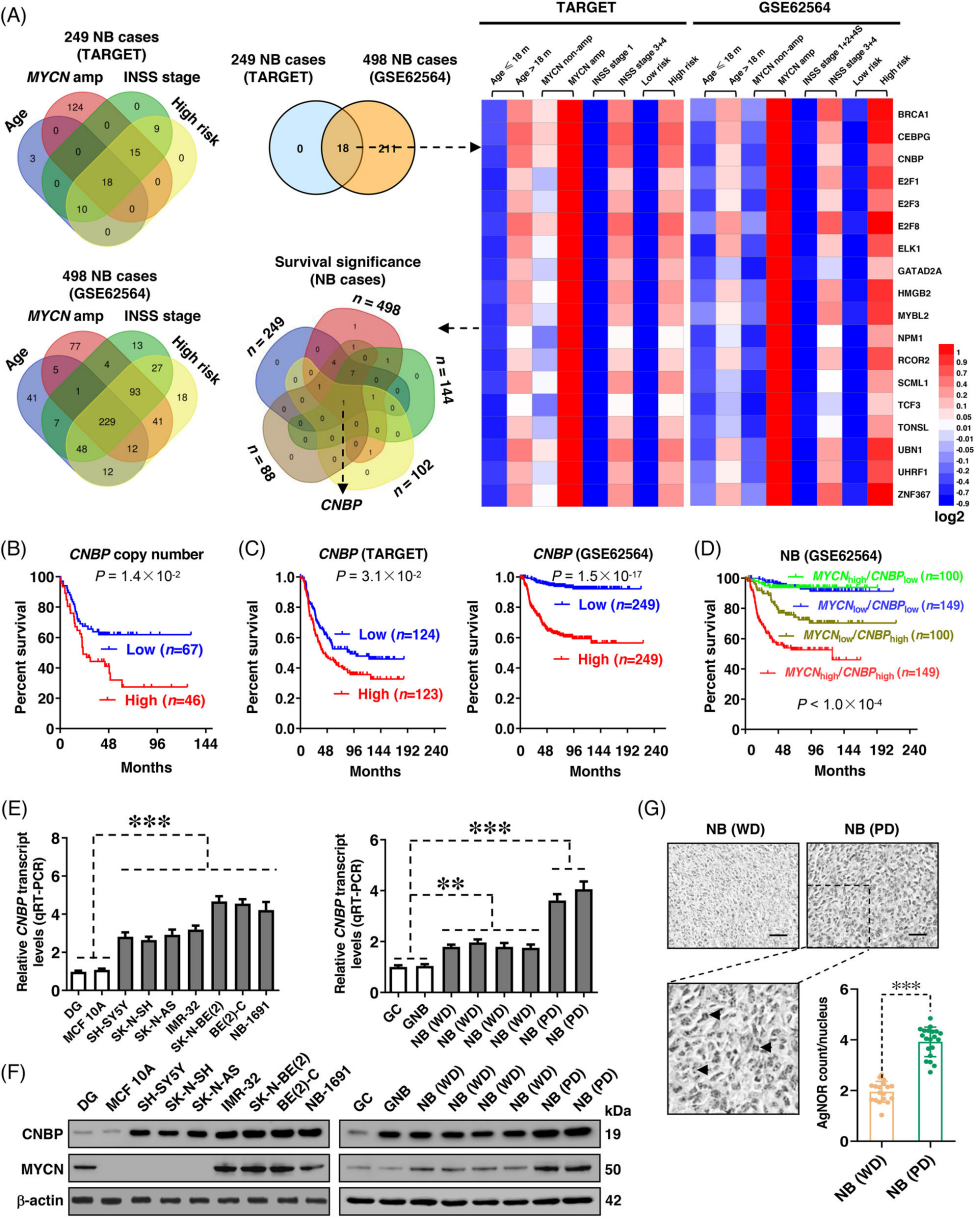
Cellular nucleic acid‐binding protein (*CNBP*) is associated with poor prognosis and clinical progression of neuroblastoma (NB). (A) Venn diagram (left and middle upper panels) and heatmap (right panel) revealing the identification of transcriptional regulators associated with older age at diagnosis (>18 months), *MYCN* amplification, advanced International Neuroblastoma Staging System (INSS) stages and high risk of 249 (TARGET) and 498 (GSE62564) NB cases. Venn diagram (middle lower panel) indicating further overlapping analysis for survival significance of identified transcriptional regulators in 249 (TARGET), 498 (GSE62564), 144 (gencode19), 102 (GSE3446) and 88 (GSE16476) NB patients. (B) Kaplan–Meier plots showing event‐free survival curves of 113 NB patients with high or low *CNBP* copy number (cutoff value = 0.992) derived from Oncogenomics database (https://omics‐oncogenomics.ccr.cancer.gov/cgi‐bin/JK). (C) Kaplan–Meier curves indicating the survival of 249 (TARGET, cutoff value = 10.01) and 498 (GSE62564, cutoff value = 6.82) NB patients with high or low *CNBP* expression. (D) Kaplan–Meier curves showing the survival of patients with high or low expression of *MYCN* (cutoff value = 5.76) and *CNBP* (cutoff value = 6.82) in 498 NB cases (GES62564). (E and F) Real‐time quantitative reverse transcription‐polymerase chain reaction (qRT‐PCR, E, normalised to *β‐actin*, *n* = 6) and western blot (F) assays indicating the expression of *CNBP* or *MYCN* in normal dorsal ganglia (DG), normal cell line (MCF 10A), NB cell lines without (SH‐SY5Y, SK‐N‐SH, SK‐N‐AS) or with *MYCN* amplification (IMR32, SK‐N‐BE(2), BE(2)‐C, NB‐1691), gangliocytoma (GC), ganglioneuroblastoma (GNB) or NB tissues (*n* = 21). (G) Representative images and quantification of argyrophilic nucleolar organiser region (AgNOR) staining assay showing the localisation (arrowheads) and number of nucleolar organiser region (NOR) dots within well differentiated (WD, *n* = 18) or poorly differentiated (PD, *n* = 20) NB tissues. Scale bars: 100 µm. Fisher's exact test for overlapping analysis in (A); log‐rank test in (B–D); one‐way analysis of variance (ANOVA) with Bonferroni's multiple comparison test in (E); unpaired two‐sided Student's *t* test in (G). ^**^ *p <* .01, ^***^ *p <* .001 versus DG, GC or NB (WD). Data are shown as mean ± standard error of the mean (s.e.m.) (error bars) and representative of three independent experiments in (E–G).

For discovering important transcriptional regulators of NB, we first analysed the differentially expressed transcriptional regulators in 249 (TARGET) NB cases with varied status of age (<18 months vs. >18 months), *MYCN* amplification (with vs. without), clinical stages of International Neuroblastoma Staging System (INSS, 1+2+4S vs. 3+4) or risk classification (low vs. high). As shown in Figure 1A, 18 transcriptional regulators were noted to be consistently associated with older age at diagnosis (>18 months), *MYCN* amplification, advanced INSS stage and high risk in 249 NB cases (Table S1). Similarly, we found 229 transcriptional regulators associated with these features in another independent cohort of 498 (GSE62564) NB cases (Figure 1A and Table S1). By overlapping analysis of results derived from these two independent cohorts, 18 transcriptional regulators were identified as candidates essential for aggressiveness of NB (Figure 1A). By analysing their survival significance, only *CNBP* was found to be associated with poor survival in 249 (TARGET), 498 (GSE62564), 144 (gencode19), 102 (GSE3446) and 88 (GSE16476) NB patients (Figure 1A and Table S1). In 113 NB cases derived from the Oncogenomics database (Table S2), high copy number of *CNBP* gene was substantially related to low event‐free survival probability (*P* = 1.4 × 10^−2^, Figure 1B). The *CNBP* levels were linked to worse alive status in 249 (TARGET, *P* = 3.1 × 10^−2^), 498 (GSE62564, *P* = 1.5 × 10^−17^), 144 (gencode19, *P* = 1.7 × 10^−4^), 102 (GSE3446, *P* = 1.5 × 10^−2^) and 88 (GSE16476, *P* = 2.4 × 10^−2^) patients suffering from NB (Figures 1C and S1A), while worse survival was observed in patients with up‐regulation of both *MYCN* and *CNBP* (Figure 1D). Elevated *CNBP* expression was detected in NB specimens or cultured cell lines (Figure 1E,F). There was up‐regulation of *Cnbp* in hyperplastic sympathetic ganglia of TH‐*MYCN* transgenic mice (Figure S1B). Notably, ectopic expression of *MYCN* led to up‐regulation of *CNBP* in both *MYCN*‐amplified and non‐amplified cells (IMR‐32 and SH‐SY5Y, Figure S1C). Although knockdown of *MYCN* decreased the levels of *CNBP* in *MYCN*‐amplified IMR‐32 cells (Figure S1C), *CNBP* expression was not associated with that of *MYCN* in NB cell lines without *MYCN* amplification (Figure 1F). Moreover, *MYCN* levels were unaffected by *CNBP* within these NB cells (Figure S1C). In multivariate analysis, *CNBP* expression served as an independent prognostic indicator in 498 (GSE62564) NB cases (Table S3). Immunohistochemical staining indicated higher nuclear CNBP expression in NB specimens with poor differentiation, accompanied by more nucleolar organiser region (NOR) dots, when compared to those with good differentiation (Figures S1D and 1G). These results suggested that *CNBP* was related to poor prognosis and clinical progression of NB.

### 2.2 *CNBP* facilitates aggressive behaviours of NB cells

**FIGURE 2:**
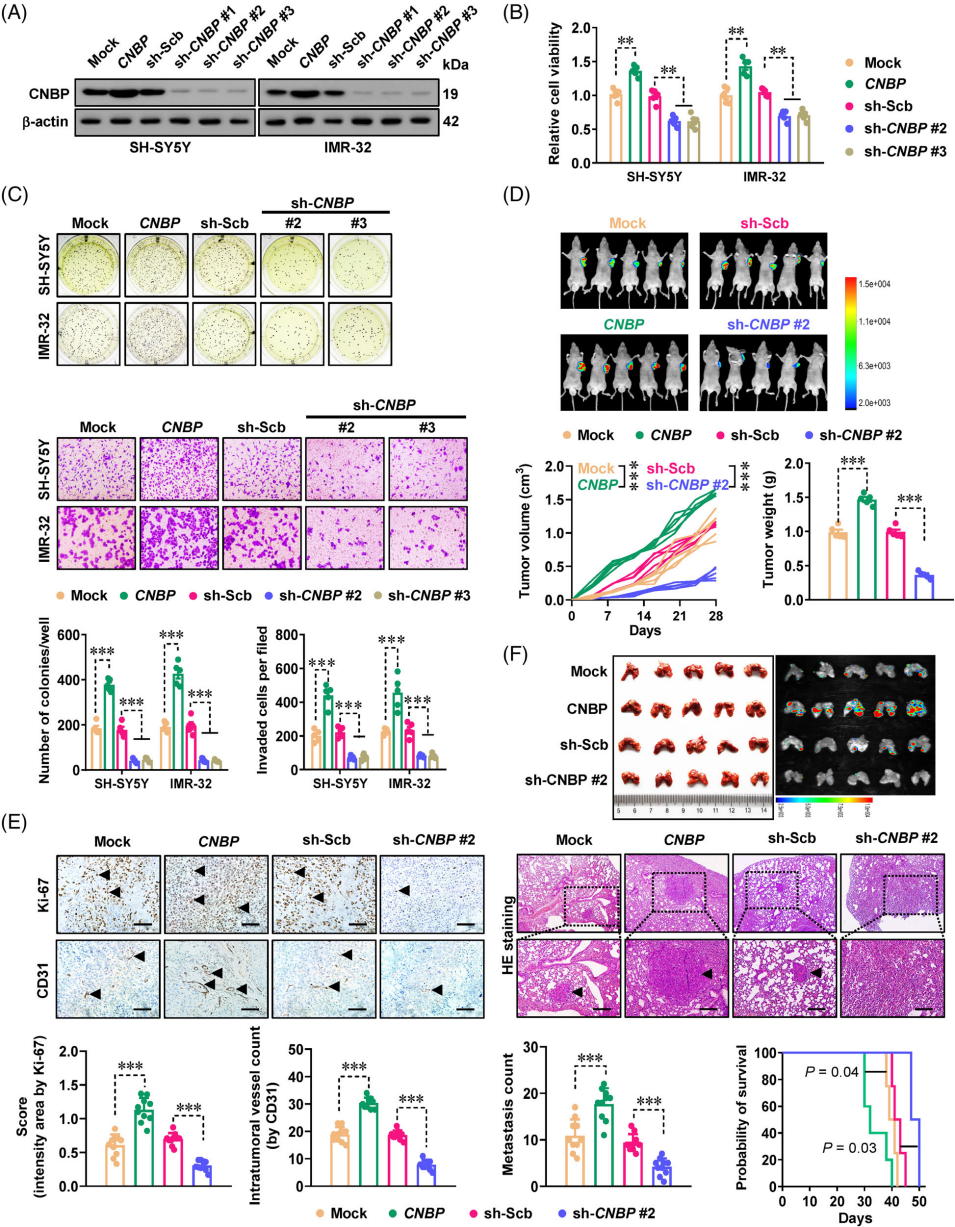
Cellular nucleic acid‐binding protein (*CNBP*) facilitates growth and aggressiveness of neuroblastoma (NB) cells in vitro and in vivo. (A) Western blot assay indicating the expression of CNBP in SH‐SY5Y and IMR‐32 cells stably transfected with empty vector (mock), *CNBP*, scramble shRNA (sh‐Scb) or sh‐*CNBP*. (B) 2‐(4,5‐Dimethyltriazol‐2‐yl)−2,5‐diphenyl tetrazolium bromide (MTT) colorimetric assay depicting the change in viability of SH‐SY5Y and IMR‐32 cells stably transfected with mock, *CNBP*, sh‐Scb or sh‐*CNBP* (*n* = 5 per group). (C) Representative images (upper panel) and quantification (lower panel) of soft agar and Matrigel invasion assays showing the anchorage‐independent growth and invasion of SH‐SY5Y and IMR‐32 cells stably transfected with mock, *CNBP*, sh‐Scb, sh‐*CNBP* #2 or sh‐*CNBP* #3 (*n* = 5 per group). (D) In vivo imaging, growth curve, and weight at the end points of xenograft tumours formed by subcutaneous injection of SH‐SY5Y cells stably transfected with mock, *CNBP*, sh‐Scb or sh‐*CNBP* #2 into dorsal flanks of nude mice (*n* = 5 for each group). (E) Representative images (upper panel) and quantification (lower panel) of immunohistochemical staining showing the intratumoural expression of Ki‐67 and CD31 (brown, arrowheads) within subcutaneous xenograft tumours of nude mice formed by SH‐SY5Y cells stably transfected with mock, *CNBP*, sh‐Scb or sh‐*CNBP* #2 (*n* = 5 for each group). Scale bars: 100 µm. (F) Representative images (top panel), haematoxylin and eosin (HE) staining (middle panel, arrowheads), quantification (bottom left panel) of lung metastatic colonisation and Kaplan–Meier curves (bottom right panel) of nude mice treated with tail vein injection of SH‐SY5Y cells stably transfected with mock, *CNBP*, sh‐Scb or sh‐*CNBP* #2 (*n* = 5 for each group). Scale bars: 100 µm. Student's *t* test and one‐way analysis of variance (ANOVA) compared the difference in (B–F). Log‐rank test for survival comparison in (F). ^**^ *p <* .01, ^***^ *p <* .001 versus mock or sh‐Scb. Data are shown as mean ± standard error of the mean (s.e.m.) (error bars) and representative of three independent experiments in (A–C).

Based on above *CNBP* expression profiling, SH‐SY5Y, IMR‐32 (with middle *CNBP* levels) and BE(2)‐C (with high *CNBP* levels) cells were selected as models for further biological studies. Within these NB cells, the expression of *CNBP* was up‐ or down‐regulated by steady transfection of *CNBP* construct, distinct short hairpin RNAs (shRNAs) against *CNBP* (sh‐*CNBP*), or nuclease‐dead clustered regularly interspaced short palindromic repeat‐associated protein 9 (dCas9) vectors^10^ (Figures 2A and S2A,B). The viability of NB cells was enhanced or diminished by over‐expression or silencing of *CNBP* (Figure 2B), accompanied by increase or decrease in 40S ribosomal subunit assembly, respectively (Figure S2C). In SH‐SY5Y cells persistently over‐expressing *CNBP*, NOR dots and protein synthesis were increased (Figure S2D,E). Notably, silencing of *CNBP*‐induced apoptosis of SH‐SY5Y (wild‐type *p53*) cells, without significant changes in cell cycle phases (Figure S2F,G). Meanwhile, there was significant G_2_/M cell cycle arrest, but no changes of apoptosis, in BE(2)‐C (mutant *p53*) cells with steady transfection of sh‐*CNBP* (Figure S2F,G), which was consistent with previous findings that inhibition of ribosome biogenesis induces apoptosis or G_2_/M arrest in wild‐type or mutant *p53* NB cells, respectively.^11^ In addition, in vitro proliferative and invasive behaviours of SH‐SY5Y and IMR‐32 cells were also increased or repressed upon steady transfection of *CNBP* or sh‐*CNBP*, respectively (Figure 2C). Consistently, steady up‐ or down‐regulation of *CNBP* caused a significant increase or decrease in volume, weight, proliferative index and microvessel density of xenograft tumours generated by subcutaneous injection of SH‐SY5Y cells into nude mice (Figures 2D,E and S2H). Meanwhile, intravenous administration of NB cells with steady *CNBP* over‐expression resulted in more lung metastases and worse survival of nude mice (Figure 2F). Conversely, nude mice treated with NB cells stably knocking down *CNBP* presented fewer lung metastatic colonies and higher survival possibility (Figure 2F). These findings revealed that *CNBP* facilitated aggressive behaviours of NB cells.

### 2.3 KPNB1 facilitates nuclear translocation of CNBP to interact with SMARCC2 in liquid condensates

**FIGURE 3:**
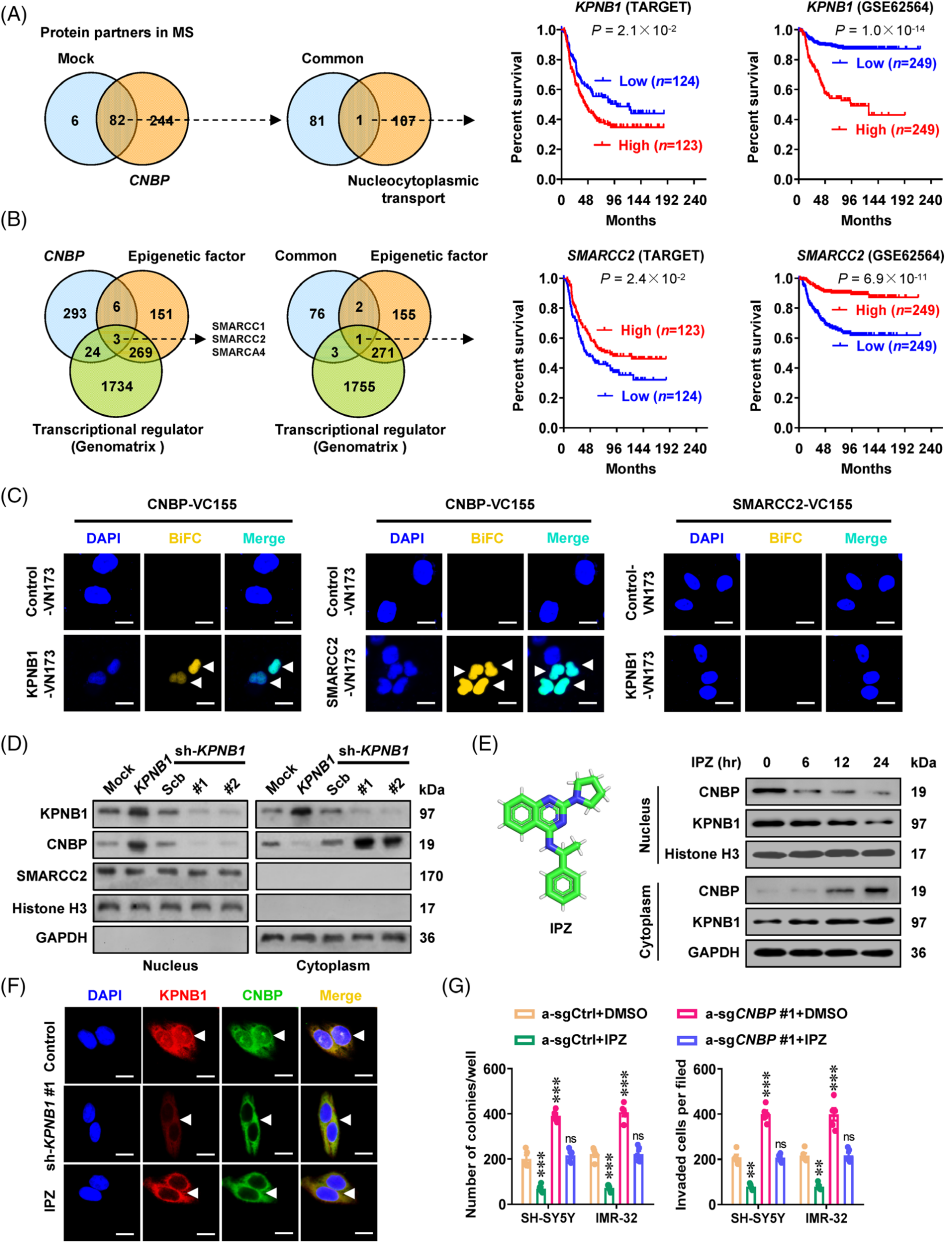
Karyopherin subunit beta 1 (*KPNB1*) facilitates nuclear translocation and oncogenic roles of cellular nucleic acid‐binding protein (*CNBP*). (A and B) Venn diagram (left panel) assays revealing the overlapping analysis of proteins pulled down by CNBP antibody from lysates of IMR‐32 cells stably transfected with empty vector (mock) or *CNBP*, and comprehensive analysis with nucleocytoplasmic transporters, epigenetic factors and transcriptional regulators derived from AmiGO2 (http://amigo.geneontology.org), EpiFactors (https://epifactors.autosome.ru/) or Genomatrix (http://www.genomatix.de) databases. Kaplan–Meier curves (right panel) indicating the survival of patients with high or low expression of *KPNB1* (cutoff values = 10.46 and 7.80) or *SMARCC2* (cutoff values = 9.42 and 7.57) in 249 (TARGET) and 498 (GES62564) neuroblastoma (NB) cases. (C) Bimolecular fluorescence complementation (BiFC) assay showing the interaction of CNBP with KPNB1 or SMARCC2 (arrowheads) in SH‐SY5Y cells co‐transfected with their respective constructs. Scale bars: 10 µm. (D) Western blot assay indicating the cytoplasmic and nuclear accumulation of CNBP in IMR‐32 cells stably transfected with mock, *KPNB1*, scramble shRNA (sh‐Scb) or sh‐*KPNB1*. (E) Western blot assay showing the cytoplasmic and nuclear levels (right panel) of CNBP in IMR‐32 cells treated with importazole (IPZ, 20 µmol L^−1^, left panel) for different time points as indicated. (F) Immunofluorescence assays revealing the cytoplasmic and nuclear levels of KPNB1 and CNBP (arrowheads) in IMR‐32 cells, and those stably transfected with sh‐*KPNB1* #1 or treated with IPZ (20 µmol L^−1^) for 24 h. Scale bars: 10 µm. (G) Quantification of soft agar and Matrigel invasion assays showing the anchorage‐independent growth and invasion capability of SH‐SY5Y and IMR‐32 cells transfected with scramble CRISPRa (CRISPRa‐Scb) or CRISPRa‐*CNBP* #1, and those treated with dimethylsulfoxide (DMSO) or IPZ (20 µmol L^−1^) for 24 h. Fisher's exact test for overlapping analysis in (A and B). Log‐rank test compared the survival difference in (A and B). One‐way analysis of variance (ANOVA) with Bonferroni's multiple comparison test in (G). ^**^ *p <* .01, ^***^ *p <*  .001 versus a‐sgCtrl + DMSO. ns, non‐significant. Data are shown as mean ± standard error of the mean (s.e.m.) (error bars) and representative of three independent experiments in (C–G).

To determine CNBP‐binding partners, CNBP antibody pulled down proteins from cellular lysates were subjected to non‐quantitative proteomic analysis (Figures 3A and S3A and Table S4). There were 88 proteins binding to endogenous CNBP in IMR‐32 cells, 82 of which were consistently detected following stable transfection of *CNBP* (Figure 3A). Among them, KPNB1 was essential for nucleocytoplasmic transport (Figure 3A), while SMARCC2 was one subunit of SWI/SNF complex (Figure 3B). Notably, high *KPNB1* expression (*P* = 2.1 × 10^−2^ and 1.0 × 10^−14^) or low *SMARCC2* levels (*P* = 2.4 × 10^−2^ and 6.9 × 10^−11^) were linked to poor outcome of NB patients in public datasets (TARGET and GSE62564, Figure 3A,B). Endogenous physical interaction of CNBP with KPNB1 or SMARCC2 protein was validated in NB cells (Figure S3B). Additionally, bimolecular fluorescence complementation (BiFC) experiment revealed that in IMR‐32 cells, CNBP interacted with either KPNB1 or SMARCC2, while no interaction was observed between KPNB1 and SMARCC2 (Figure 3C). Without affecting the expression of *CNBP* or *SMARCC2*, ectopic expression of *KPNB1* boosted nuclear translocation of CNBP in NB cells (Figures 3D and S3C). Meanwhile, genetic knockdown or pharmacological inhibition of *KPNB1* using importazole (IPZ)^12^ led to cytoplasmic accumulation of CNBP in IMR‐32 cells (Figures 3D–F and S3D). In particular, IPZ treatment reduced the increase in proliferation and invasion of NB cells induced by *CNBP* activation (Figures 3G and S3E).

**FIGURE 4:**
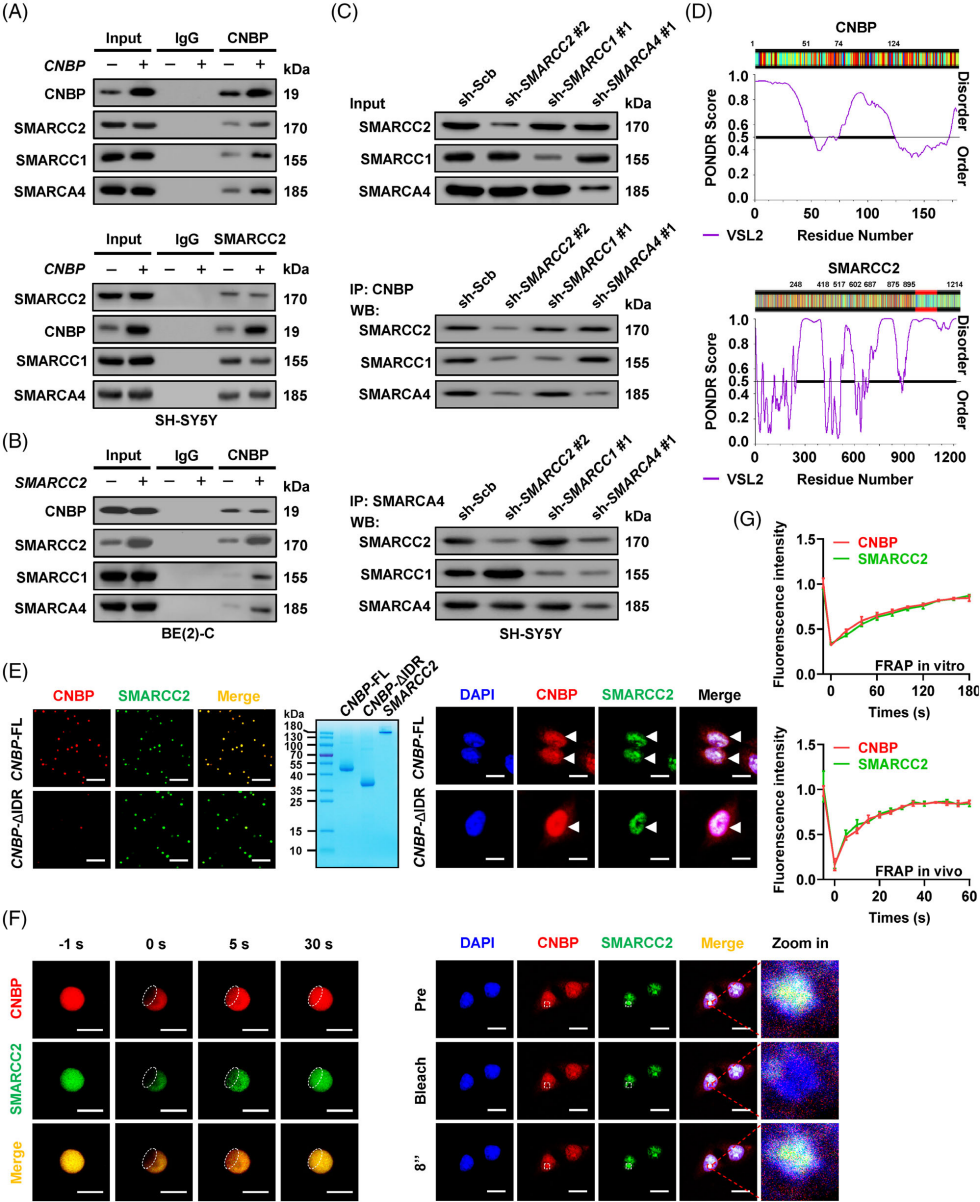
Cellular nucleic acid‐binding protein (CNBP) interacts with switch/sucrose‐nonfermentable (SWI/SNF) complex via SMARCC2 in liquid condensates. (A and B) Co‐immunoprecipitation (Co‐IP) and western blot assays indicating the interaction of CNBP with SWI/SNF complex subunits SMARCC2, SMARCC1 or SMARCA4 in SH‐SY5Y or BE(2)‐C cells stably transfected with empty vector (mock), *CNBP* or *SMARCC2*. (C) Co‐IP and western blot assays showing the interaction among CNBP, SMARCC2, SMARCC1 and SMARCA4 in SH‐SY5Y cells stably transfected with scramble shRNA (sh‐Scb), sh‐*SMARCC2* #2, sh‐*SMARCC1* #1 or sh‐*SMARCA4* #1. (D) Intrinsically disordered region (IDR) within CNBP and SMARCC2 proteins analysed by PONDR (http://www.pondr.com/) program. (E) Fluorescence imaging assay indicating the condensate formation of recombinant full‐length (FL) or IDR‐deficient CNBP‐mCherry and SMARCC2‐EGFP proteins (left panel) with purity detected by SDS‐PAGE and Coomassie blue staining (middle panel), and that of CNBP and SMARCC2 in SH‐SY5Y cells stably transfected with *CNBP* construct (right panel, arrowheads). Scale bars: 10 µm. (F and G) Representative images (F) and quantification (G) of fluorescence recovery after photobleaching (FRAP) assay showing the exchange kinetics of CNBP‐mCherry and SMARCC2‐EGFP within condensates, and those of CNBP and SMARCC2 in SH‐SY5Y cells stably transfected with *CNBP* construct. Scale bars: 10 µm. Data are shown as representative of three independent experiments in (A–C) and (E–G).

Since there were also SMARCA4 and SMARCC1, established SWI/SNF core subunits (Figure S4A), in proteins pulled down by CNBP antibody from IMR‐32 cells stably over‐expressing *CNBP* (Figure 3B), we further investigated the interaction of CNBP with these SWI/SNF subunits. Steady transfection or knockdown of *CNBP* promoted or inhibited the interaction of CNBP with SMARCC2, SMARCC1 or SMARCA4 in SH‐SY5Y and IMR‐32 cells (Figures 4A and S4B). When *SMARCC2* was over‐expressed or knocked down, the interaction of CNBP with SMARCC1 or SMARCA4 in NB cells was increased or attenuated (Figures 4B,C and S4C,D). The physical interaction between CNBP and SMARCC2 was unaffected by silencing of *SMARCC1* or *SMARCA4* (Figures 4C and S4E). Alternatively, *SMARCC2* silencing promoted the interaction of SMARCA4 with SMARCC1 in NB cells (Figure 4C). Notably, arginine–glycine–glycine (RGG) domain (22–42 amino acids [aa]), but not zinc finger domains, of CNBP protein mediated its binding to SMARCC2 (Figure S4F,G). Meanwhile, SANT domain (596–647 aa), rather than Swirm domain (424–521 aa) or C170 domain (647–1215 aa), within SMARCC2 protein was required for its interaction with CNBP, which was also demonstrated by in vitro binding test using glutathione S‐transferase (GST)‐tagged CNBP or His‐tagged SMARCC2 proteins (Figure S4F,G). Since RGG domain of CNBP protein was discovered to locate within intrinsically disordered regions (IDRs) via Preditor of Natural Disordered Regions program^13^ (Figure 4D), the potential liquid–liquid phase separation (LLPS) of CNBP and SMARCC2 protein was further investigated. Fluorescence imaging assay indicated that recombinant CNBP‐mCherry and SMARCC2‐EGFP proteins (purity > 90%) assembled into condensates in vitro, with similar compartmentation in SH‐SY5Y cells, while deletion of IDR abolished the LLPS of CNBP in vitro and in vivo (Figure 4E). To explore their liquid‐like features, fluorescence recovery after photobleaching (FRAP) assay was performed, which indicated rapid exchange kinetics of CNBP and SMARCC2 within condensates (Figure 4F,G). These data indicated that KPNB1 facilitated nuclear translocation of CNBP to interact with SMARCC2 in liquid condensates.

### 2.4 Phase separation of CNBP modulates SWI/SNF activity to facilitate gene expression essential for 40S ribosomal subunit assembly

**FIGURE 5:**
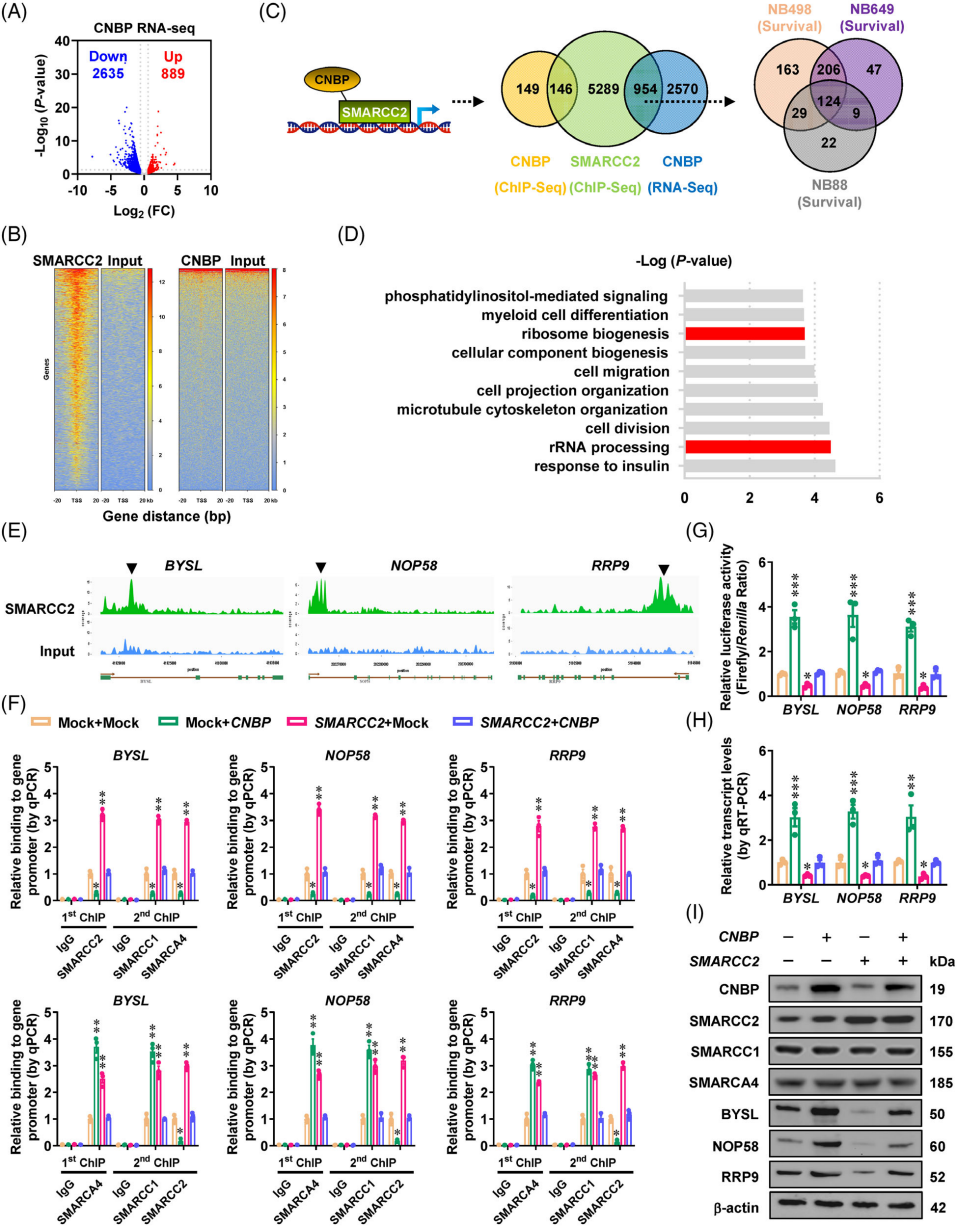
Cellular nucleic acid‐binding protein (CNBP) facilitates switch/sucrose‐nonfermentable (SWI/SNF) target gene expression essential for 40S ribosomal subunit assembly. (A) Volcano plots of RNA sequencing (RNA‐seq) assay revealing the alteration of gene expression (fold change > 1.5, false discovery rate < 0.05) in IMR‐32 cells stably transfected with empty vector (mock) or *CNBP*. (B) Chromatin immunoprecipitation sequencing (ChIP‐seq) assay indicating endogenous enrichment of SMARCC2 or CNBP on target genes in IMR‐32 cells. (C) Schematic depiction (left panel) and Venn diagram (middle and right panels) showing the target genes of *CNBP* or *SMARCC2* in indicated action modes, and those associated with survival of 498 (GSE62564), 649 (GSE45547) and 88 (GSE16476) neuroblastoma (NB) patients. (D) DAVID analysis of 124 *SMRCCC2* target genes with significant association with survival of NB patients. (E) ChIP‐seq peak indicating SMARCC2 enrichment on promoter regions of target genes *BYSL*, *NOP58* and *RRP9*. (F) By using first antibody specific for SMARCC2 or SMARCA4, sequential ChIP and qPCR assays indicating relative enrichment of SMARCC2, SMARCC1 or SMARCA4 (normalised to input) on promoter regions of *BYSL*, *NOP58* and *RRP9* in IMR‐32 cells stably transfected with mock, *SMARCC2* or *CNBP* (*n* = 5). (G–I) Dual‐luciferase (G), real‐time quantitative reverse transcription‐polymerase chain reaction (qRT‐PCR, H) and western blot (I) assays indicating the promoter activity, transcript and protein levels of target genes *BYSL*, *NOP58* and *RRP9*, as well as the expression of SMARCC1 and SMARCA4, in IMR‐32 cells stably transfected with mock, *CNBP* or *SMARCC2*. Fisher's exact test for overlapping analysis in (C). One‐way analysis of variance (ANOVA) with Bonferroni's multiple comparison test in (F–H). ^*^ *p <*  .05, ^**^ *p <*  .01, ^***^ *p <* .001 versus mock + mock. Data are shown as mean ± standard error of the mean (s.e.m.) (error bars) and representative of three independent experiments in (F–I).

To determine target genes of *CNBP* or *SMARCC2*, RNA sequencing revealed that ectopic expression of *CNBP* led to significant expression alteration of 3524 genes in IMR‐32 cells (Figure 5A and Table S5). In chromatin immunoprecipitation sequencing (ChIP‐seq) assay, there were 6389 and 295 genes with endogenous SMARCC2 or CNBP‐binding peaks, respectively (Figure 5B). Based on comprehensive analysis of possible action modes, 954 genes were identified as top ranking targets uniquely recognised by SMARCC2, with CNBP acting as a co‐factor (Figures 5C and S5A). In three separate datasets (GSE62564, GSE45547, GSE16476), 124 of them had consistent relationship with NB patients survival (Figures 5C and S5B), while DAVID analysis (https://david‐d.ncifcrf.gov) revealed ribosome biogenesis as an important involved pathway (Figure 5D and Table S6), mainly participated by bystin like (*BYSL*), NOP58 ribonucleoprotein (*NOP58*) and ribosomal RNA processing 9 (*RRP9*, Figure 5E). Notably, ectopic expression of *CNBP* or its IDR‐deficient form abolished or facilitated the enrichment of SMARCC2 on these target genes (Figure S5C), resulting in elevated or decreased promoter activity and transcript levels of *BYSL*, *NOP58* and *RRP9*, respectively (Figure S5D,E). To further reveal the effects of *CNBP* and SWI/SNF subunits on target gene expression, sequential ChIP and quantitative PCR (qPCR) assays were performed using first antibody specific for SMARCC2, SMARCC1 or SMARCA4, respectively. Ectopic expression of *CNBP* reduced the enrichment of SMARCC2/SMARCC1/SMARCA4 subunits, but increased the alternative binding of SMARCC1/SMARCA4 binary complex, on promoter regions of *BYSL*, *NOP58* and *RRP9*, which was abolished by transfection of *SMARCC2* (Figures 5F and S5F). Notably, knockdown of *SMARCC1* or *SMARCA4* attenuated the up‐regulation of target genes induced by ectopic *CNBP* expression (Figure S5G,H). In public datasets of 249 (TARGET) and 498 (GSE62564) NB patients, elevated levels of *SMARCC1* or *SMARCA4* were linked to worse survival (Figure S5I). Accordingly, up‐regulation of *SMARCC2* caused a decrease in promoter activation, transcript levels, as well as protein expression of *BYSL*, *NOP58* or *RRP9* in IMR‐32 cells, which was reversed by over‐expression of *CNBP* (Figure 5G–I). Rather, the levels of previously recognised SWI/SNF target genes *c‐Myc* or *cyclin D1* ^14^, ^15^, ^16^ were not affected by *CNBP*, *SMARCC2*, *SMARCC1* or *SMARCA4* in SH‐SY5Y cells (Figures S5J,K and S12). In addition, knockdown of *BYSL*, *NOP58* or *RRP9* restored the alteration in their expression and aggressive features of NB cells following *CNBP* activation (Figure S6A–E).

**FIGURE 6:**
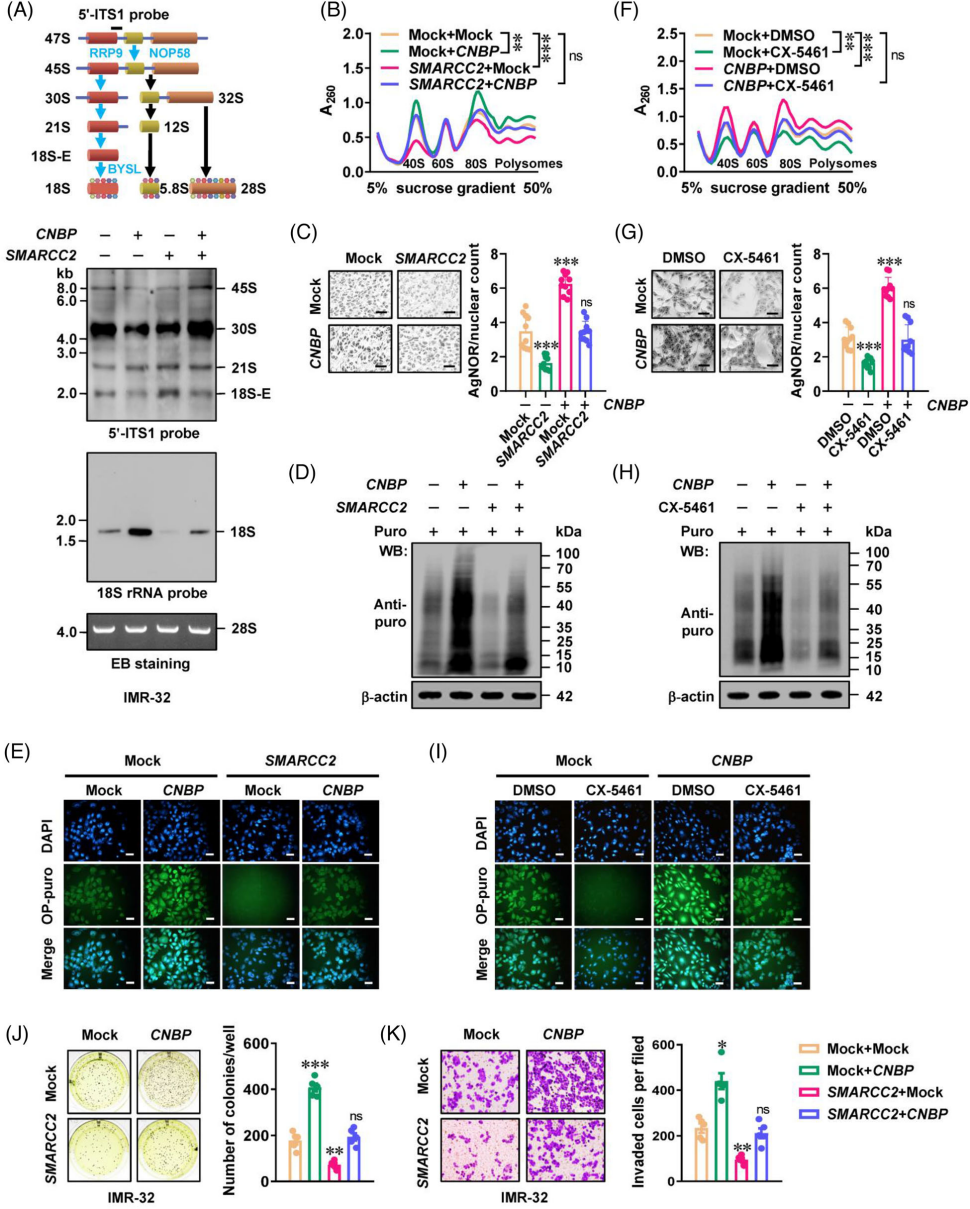
Cellular nucleic acid‐binding protein (*CNBP*) represses tumour suppressive roles of *SMARCC2* in ribosome biogenesis. (A) Schematic diagram (upper panel) indicating ribosomal RNA (rRNA) processing in human cells. Northern blot using 5′‐ITS1 or 18S rRNA probe (lower panels) revealing the amount of 18S rRNA precursors in IMR‐32 cells stably transfected with empty vector (mock), *SMARCC2* or *CNBP*. The 28S rRNA was shown as loading control. (B–E) Sucrose gradient sedimentation (B), argyrophilic nucleolar organiser region (AgNOR) staining (C, *n* = 5), puromycin incorporation (D), and OP‐puro incorporation (E) assays showing the ribosomal subunit assembly, nucleolar organiser region (NOR) dots, nascent protein synthesis and protein production in IMR‐32 cells stably transfected with mock, *SMARCC2* or *CNBP*. (F–I) Sucrose gradient sedimentation (F), AgNOR staining (G, *n* = 5), puromycin incorporation (H) and OP‐puro incorporation (I) assays indicating the ribosomal subunit assembly, nucleolar NOR dots, nascent protein synthesis and protein production in IMR‐32 cells stably transfected with mock or *CNBP*, and those treated with dimethylsulfoxide (DMSO) or CX‐5461 (5 µmol L^−1^) for 48 h (*n* = 4). (J and K) Representative images (left panel) and quantification (right panel) of soft agar (J) and Matrigel invasion (K) assays showing the anchorage‐independent growth and invasion of IMR‐32 cells stably transfected with mock or *SMARCC2*, and those co‐transfected with *CNBP* (*n* = 5). One‐way analysis of variance (ANOVA) with Bonferroni's multiple comparison test in (B, C, F, G, J and K). ^*^ *p <* .05, ^**^ *p <* .01, ^***^ *p <* .001 versus mock + mock. Data are shown as mean ± standard error of the mean (s.e.m.) (error bars) and representative of three independent experiments in (A–K).

Consistently, persistent over‐expression of *CNBP* facilitated the 18S rRNA processing from 18S‐E pre‐rRNA in IMR‐32 cells (Figure 6A), accompanied by increased abundance of 40S ribosomal subunit, 80S ribosomal subunit and polysomes (Figure 6B). Meanwhile, ectopic expression of *SMARCC2* restored the changes in 18S rRNA levels, ribosome biogenesis, NORs dots and protein synthesis induced by *CNBP* (Figures 6A–E and S7A). Treatment with CX‐5461, an established inhibitor of ribosome biogenesis,^17^ repressed the ribosomal subunit assembly (Figure 6F), and rescued the increase in NORs dots, nascent protein production and protein synthesis of NB cells induced by *CNBP* (Figures 6G–I and S7A). Stable over‐expression of *CNBP* caused an increase in proliferative or invasive features of NB cells, which was prevented upon *SMARCC2* transfection or CX‐5461 treatment (Figures 6J,K and S7B,C). These results suggested that phase separation of CNBP modulated SWI/SNF activity to facilitate gene expression essential for 40S ribosomal subunit assembly.

### 2.5 *CNBP* exerts oncogenic functions via repressing SMARCC2 activity

**FIGURE 7:**
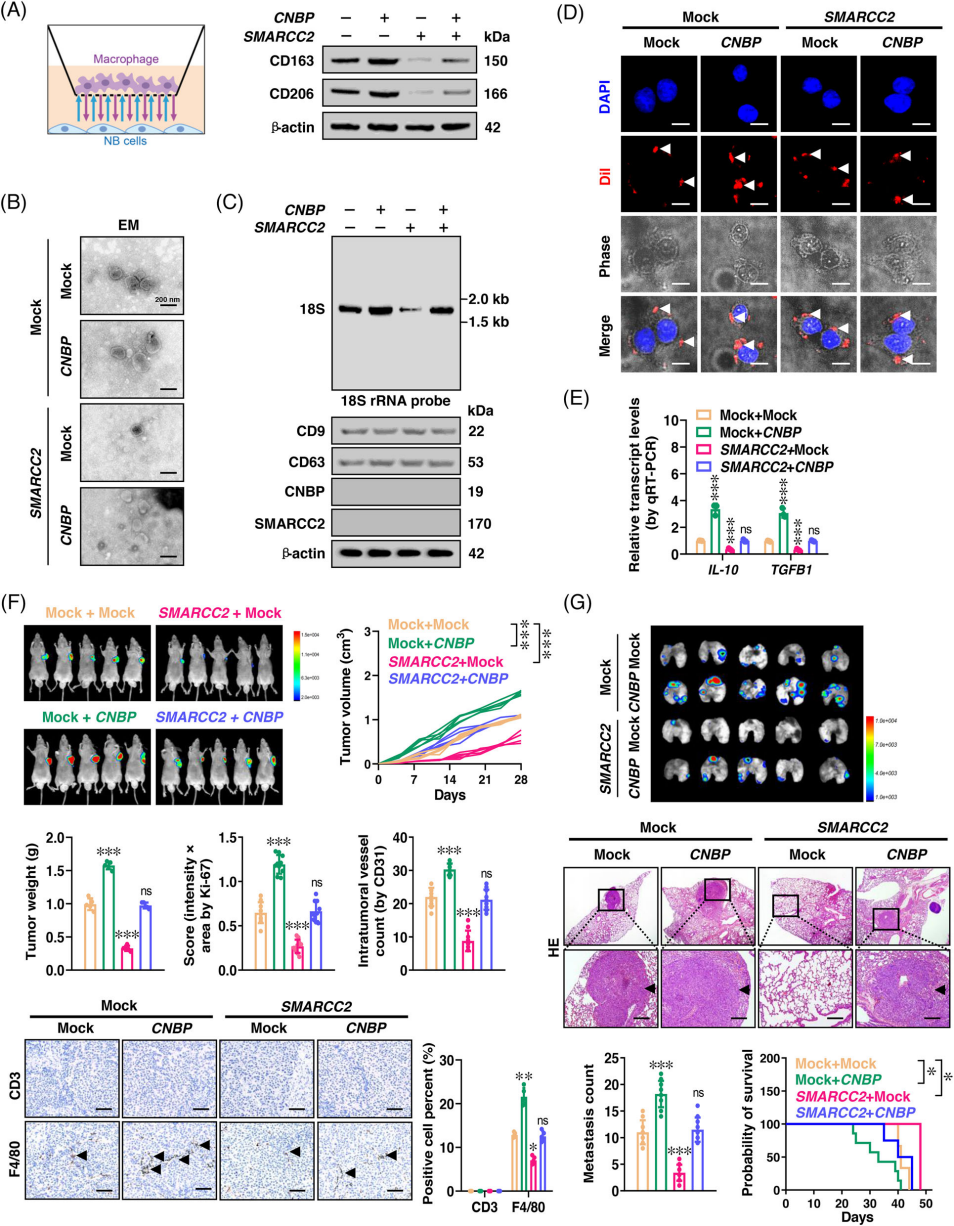
Cellular nucleic acid‐binding protein (*CNBP*) facilitates extracellular vesicle‐mediated 18S ribosomal RNA (rRNA) delivery and M2 macrophages polarization via repressing SMARCC2 activity. (A) Schematic illustration (left panel) and western blot (right panel) indicating the polarisation of CD163‐ and CD206‐positive M2 macrophages from Tohoku Hospital Pediatrics‐1 (THP‐1) cells co‐cultured with IMR‐32 cells stably transfected with empty vector (mock), *SMARCC2* or *CNBP*. (B and C) Electron microscopic observation (B), Northern blot and western blot (C) assays showing extracellular vesicles (EVs) extracted from IMR‐32 cells stably transfected with mock, *SMARCC2* or *CNBP*. (D) Confocal images indicating uptake of Dil‐labelled EVs (red color, arrowheads) extracted from IMR‐32 cells stably transfected with mock, *SMARCC2* or *CNBP* into M2 macrophages. Scale bars: 10 µm. (E) Real‐time quantitative reverse transcription‐polymerase chain reaction (qRT‐PCR) assay showing the transcript levels (normalised to *β‐actin*) of secretory macrophage markers interleukin‐10 (*IL‐10*) and transforming growth factor beta‐1 (*TGFB1*) in THP‐1 cells treated with EVs extracted from IMR‐32 cells stably transfected with mock, *SMARCC2* or *CNBP* (*n* = 5). (F) In vivo imaging, growth, weight at the end points, Ki‐67 and CD31 expression, F4/80‐positive macrophages (brown, arrowheads) of subcutaneous xenograft tumours formed by injection of IMR‐32 cells stably transfected with mock or *SMARCC2*, and those co‐transfected with *CNBP* into the dorsal flanks of nude mice (*n* = 5 for each group). Scale bars: 100 µm. (G) In vivo imaging, haematoxylin and eosin staining (arrowheads), quantification of lung metastatic colonisation and Kaplan–Meier curves of nude mice treated with tail vein injection of IMR‐32 cells stably transfected with mock or *SMARCC2*, and those co‐transfected with *CNBP* (*n* = 5 for each group). Scale bars: 100 µm. One‐way analysis of variance (ANOVA) with Bonferroni's multiple comparison test in (E–G). Log‐rank test for survival comparison in (G). ^*^ *p <* .05, ^**^ *p <* .01, ^***^ *p <* .001 versus mock + mock. Data are shown as mean ± standard error of the mean (s.e.m.) (error bars) and representative of three independent experiments in (A–E).

Since immunohistochemical staining revealed the enrichment of F4/80^+^ M2 macrophages in subcutaneous xenograft tumours formed by *CNBP* over‐expressing NB cells (Figure S7D), the impacts of *CNBP* or *SMARCC2* on interplay between NB and macrophages were further explored. During co‐culture studies, NB cells stably over‐expressing *SMARCC2* inhibited the polarization of M2 macrophages from monocyte cell line Tohoku Hospital Pediatrics‐1 (THP‐1), while ectopic expression of *CNBP* rescued this alteration (Figure 7A). We further extracted EVs from culture medium of NB cells, and validated them via electron microscopic analysis (Figure 7B), particle size assay (Figure S7E) and surface marker (CD9 and CD63) detection (Figure 7C). The 18S rRNA levels were reduced in EVs isolated from NB cells stably over‐expressing *SMARCC2*, however, ectopic expression of *CNBP* restored this change (Figure 7C). The NB cells' ability to transmit Dil‐labelled EVs to macrophages was observed via immunofluorescence assay (Figure 7D). In M2 macrophage co‐cultured with IMR‐32 cells stably over‐expressing *SMARCC2*, there was a substantial reduction in levels of transforming growth factor beta‐1 (*TGFB1*) and interleukin‐10 (*IL‐10*), which was reversed by transfection of *CNBP* (Figure 7E). As a return, M2 macrophage culture medium significantly stimulated the viability, proliferation and invasive behaviours of NB cells (Figure S7F–H). Steady transfection of *SMARCC2* led to decrease in volume, weight, proliferative index, microvessel density and M2 macrophage accumulation of subcutaneous tumours produced by IMR‐32 cells in immune‐deficient mice, and these effects were eliminated by transfection of *CNBP* (Figures 7F and S8A,B). Meanwhile, there were fewer lung metastatic counts and higher survival possibility of nude mice after intravenous injection of IMR‐32 cells with steady *SMARCC2* over‐expression, which were reversed by *CNBP* transfection (Figures 7G and S8C). These findings showed that *CNBP* suppressed *SMARCC2* activity to exert its oncogenic effects.

### 2.6 Targeting phase separation and interaction of CNBP with SMARCC2 inhibits NB progression

**FIGURE 8:**
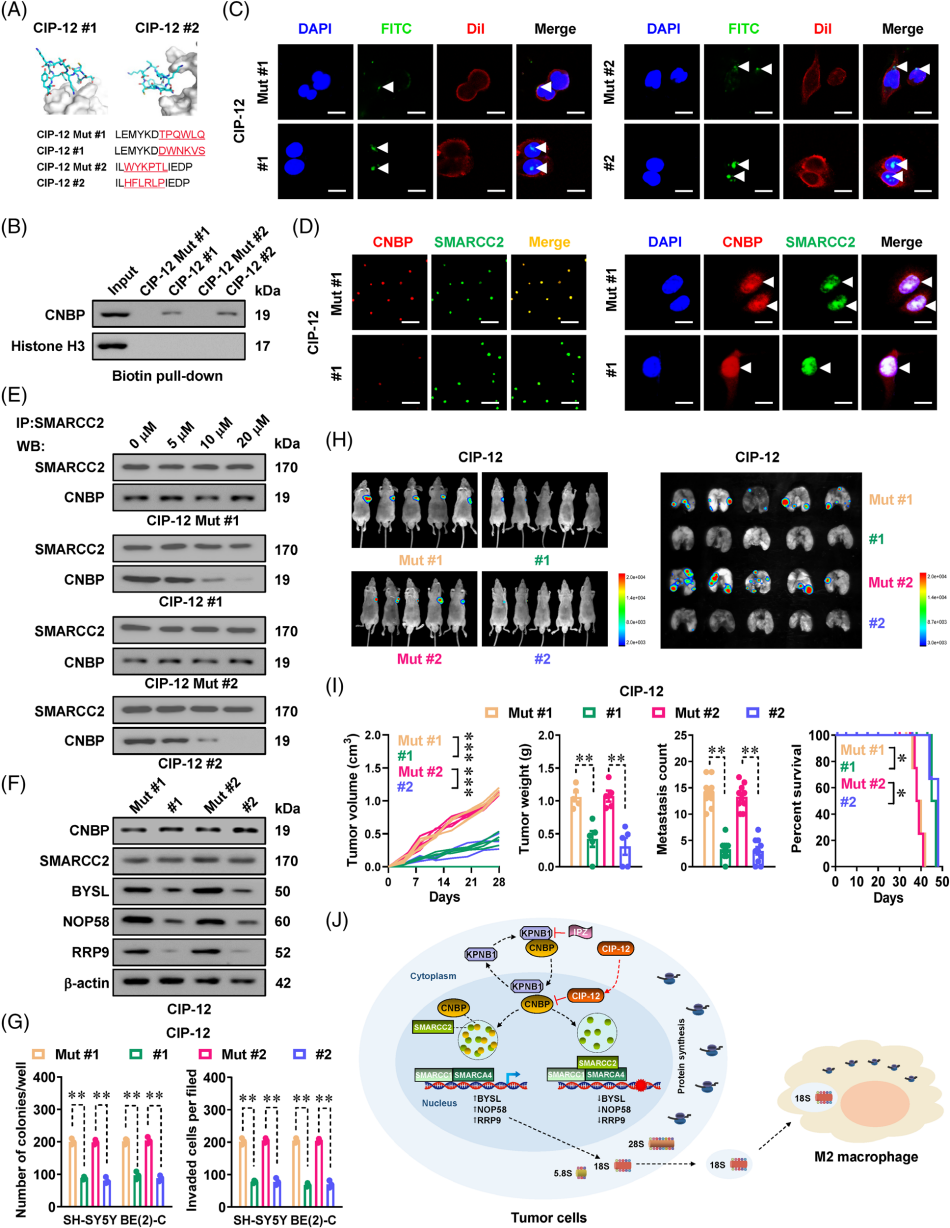
Targeting phase separation and interaction of cellular nucleic acid‐binding protein (CNBP) with SMARCC2 inhibits neuroblastoma (NB) progression. (A) Three‐dimensional structure and sequences of wild‐type or mutant (Mut) inhibitory peptide (CIP‐12) blocking interaction between CNBP and SMARCC2. (B) Biotin‐labelled peptide pull‐down and western blot assays indicating the binding of inhibitory peptides (20 µmol L^−1^) to CNBP protein within lysates of BE(2)‐C cells. (C) Confocal images showing the distribution (arrowheads) of FITC‐labelled CIP‐12 or CIP‐12 Mut (20 µmol L^−1^) within cultured BE(2)‐C cells, with nuclei and cellular membranes staining by 4′,6‐diamidino‐2‐phenylindole (DAPI) or Dil. Scale bars: 10 µm. (D) Fluorescence imaging assay revealing the condensates of recombinant CNBP‐mCherry and SMARCC2‐EGFP proteins (left panel), CNBP and SMARCC2 in BE(2)‐C cells stably transfected with *CNBP* constructs (right panel, arrowheads), and those treated with CIP‐12 or CIP‐12 Mut (20 µmol L^−1^). Scale bars: 10 µm. (E) Co‐immunoprecipitation (Co‐IP) and western blot assays indicating the interaction between CNBP and SMARCC2 in BE(2)‐C cells treated with different doses of CIP‐12 or CIP‐12 Mut for 48 h. (F) Western blot assay showing the expression of CNBP, SMARCC2 or target genes in BE(2)‐C cells treated with CIP‐12 or CIP‐12 Mut (20 µmol L^−1^) for 48 h. (G) Quantification of soft agar and Matrigel invasion assays showing the anchorage‐independent growth and invasion capability of viable SH‐SY5Y and BE(2)‐C cells pretreated with CIP‐12 or CIP‐12 Mut (20 µmol L^−1^) for 48 h (*n* = 5 per group). (H and I) In vivo imaging (H), growth curve, weight at the end points (I) of xenograft tumours formed by subcutaneous injection of BE(2)‐C cells in nude mice (*n* = 5 per group) that were subsequently treated with intravenous injection of CIP‐12 or CIP‐12 Mut (3 mg kg^−1^) as indicated. Quantification of lung metastatic colonisation and Kaplan–Meier curves (I) of nude mice (*n* = 5 per group) treated with tail vein injection of BE(2)‐C cells and CIP‐12 or CIP‐12 Mut (3 mg kg^−1^) as indicated. (J) Mechanisms underlying oncogenic roles of *CNBP*: karyopherin subunit beta 1 (KPNB1) is responsible for nuclear transport of CNBP, whereas liquid condensates of CNBP interact with SMARCC2 to selectively inhibit the activity of SMARCC2/SMARCC1/SMARCA4 subunits, resulting in increase of SMARCC1/SMARCA4 binary complex‐facilitated gene expression essential for rRNA processing and ribosome biogenesis in tumour cells, which subsequently leads to extracellular vesicle‐mediated delivery of 18S rRNA and subsequent M2 macrophages polarization. A cell‐penetrating peptide blocking phase separation and interaction of CNBP with SMARCC2 inhibits ribosome biogenesis and progression of NB. Student's *t* test and one‐way analysis of variance (ANOVA) with Bonferroni's multiple comparison test in (G and I). Log‐rank test for survival comparison in (I). ^*^ *p <* .05, ^**^ *p <* .01, ^***^ *p <* .001 versus CIP‐12 Mut. Data are shown as mean ± standard error of the mean (s.e.m.) (error bars) and representative of three independent experiments in (A–G).

Considering the significance of RGG region for interaction of CNBP with SMARCC2, we prepared cell‐penetrating peptides by using the Peptiderive website,^18^ which were designated as CNBP inhibitory peptides with a length of 12 aa (CIP‐12, Figure 8A). CIP‐12, but not the mutant control peptide (CIP‐12 Mut), pulled down endogenous CNBP from BE(2)‐C cell lysates (Figure 8B). There was accumulation of peptides within the nucleus of NB cells treated with CIP‐12, but not with CIP‐12 Mut (Figure 8C). The phase separation of CNBP, but not of SMARCC2, was abolished by CIP‐12 treatment (Figure 8D). In BE(2)‐C cells, CIP‐12 treatment abolished endogenous interaction of CNBP with SMARCC2 (Figure 8E), resulting in alteration of downstream genes (Figure 8F). CIP‐12 administration reduced the viability of BE(2)‐C cells, rather than affecting that of non‐transformed cells (Figure S9A). Additionally, CIP‐12 reduced the ribosomal subunit assembly, NOR dots, protein synthesis, proliferation and invasiveness of NB cells (Figures S9B–F and 8G). Intravenous administration of CIP‐12, but not CIP‐12 Mut, reduced the volume, weight, downstream gene expression, Ki‐67 levels, CD31‐positive microvessels, NOR dot number and M2 macrophage accumulation of BE(2)‐C‐formed subcutaneous tumours (Figures 8H,I and S10A). Intravenous treatment of CIP‐12 into nude mice also decreased the lung metastasis generated by BE(2)‐C cells, and also prolonged mice survival time (Figures 8H,I and S10B). In two public NB datasets (GSE62564 and GSE45547), *BYSL* (*P* = 1.0 × 10^−17^ and 1.8 × 10^−9^), *NOP58* (*P* = 3.4 × 10^−13^ and 2.9 × 10^−8^) or *RRP9* (*P* = 4.6 × 10^−14^ and 2.0 × 10^−13^) expression was substantially linked to worse survival of patients (Figure S11). Above all, these findings showed that blocking phase separation and interaction of CNBP with SMARCC2 inhibited NB progression.

## 3 DISCUSSION

Ribosome biogenesis is a biological process for translating mRNA into protein.^19^ Cells with vigorous growth and metabolism usually have large nucleoli and active rRNA synthesis,^19^ while hyper‐activation of ribosome biogenesis is crucial for the development and spread of tumours.^20^ Meanwhile, tumour suppressor *p53* is activated upon inhibition of ribosome biogenesis.^11^, ^21^ Herein, we discovered *CNBP* as an independent factor affecting NB outcome by driving ribosome biogenesis. Although *MYCN* facilitated the levels of *CNBP* in *MYCN*‐amplified NB cells, the mechanisms regulating high *CNBP* expression in *MYCN*‐non‐amplified NB warrant further investigation. We found that knockdown of *CNBP* inhibited ribosome biogenesis, leading to apoptosis or cell cycle G_2_/M arrest in wild‐type or mutant *p53* NB cells. Since lung metastasis is linked to a poor prognosis of NB patients,^22^ the effects of *CNBP* on aggressiveness of NB were investigated in a mouse lung metastasis model. During the study, we observed no obvious metastasis in the liver, kidney or bone marrow. However, in severe combined immunodeficiency (SCID)‐Beige mice, vein tail injection of NB cells results in metastasis in kidney, lung and liver.^23^ We believe that this difference may be due to varied immune microenvironment in host that determines the survival of tumour cells in distant organs.

Previous studies have shown that *CNBP* promotes structure rearrangement of nucleic acids in an adenosine triphosphate (ATP)‐independent way, and regulates gene expression at the transcriptional or post‐transcriptional levels.^3^ Unexpected, our results indicate that CNBP serves as a modulator of SWI/SNF subunit activity via interacting with SMARCC2. We demonstrate that nuclear transport of CNBP is mediated by KPNB1, whereas phase separation of CNBP inhibits the activity of SMARCC2/SMARCC1/SMARCA4 subunits, resulting in alternative increase of SMARCC1/SMARCA4 binary complex‐facilitated gene expression essential for 18S rRNA processing and ribosome biogenesis (Figure 8J), including *BYSL*,^24^ *NOP58* ^25^ and *RRP9*.^26^ Previous investigation reveals that *BYSL* is enhanced in breast cancer, intestinal adenocarcinomas or gastric cancer, and may promote cell proliferation by facilitating 18S rRNA processing.^27^ Meanwhile, knockdown of *BYSL* decreases the proliferation of hepatocellular carcinoma cells.^27^ NOP58, a crucial part of the box C/D snoRNP complex, participates in pre‐rRNA cleavage and maturation.^25^ In eukaryotes, RRP9 binds to U3 box C/D snoRNA, which is necessary for 18S rRNA processing and 40S subunit assembly, while genetic depletion of *RRP9* inhibits early cleavage of primary pre‐rRNA transcript and production of 18S rRNA.^26^ By gain‐ and loss‐of‐function assays, we found that *CNBP* possessed oncogenic properties via repressing SMARCC2 activity, indicating its potential value in targeting therapeutics of malignancies.

*KPNB1* is the first identified nuclear transport factor,^28^ which imports a large amount of transcriptional regulators into nucleus.^28^ Previous studies have shown that *KPNB1* expression is elevated in transformed ovarian cells, cervical cancer, ovarian cancer and breast cancer.^28^ During nuclear transport process, karyopherin protein usually requires Ran to provide energy,^28^ while blocking RanGTP binding to KPNB1 via a small molecule inhibitor (IPZ) is efficient for treatment of prostate cancer.^12^ In this study, we discovered that KPNB1 continuously imported CNBP into nucleus of NB cells, while administration of IPZ restrained this process and suppressed the aggressive behaviours of NB cells.

Phase separation, a compartmentalisation allowing for high local concentrations of proteins and nucleic acids, is crucial for gene transcription, protein homeostasis or signal transduction.^29^ For example, phase separation of polycomb repressive complex 1 and autism susceptibility candidate 2 activates gene expression,^30^ while β‐catenin condensates are essential for Wnt signalling.^31^ Meanwhile, the functions of c‐Myc or p27 are disrupted by LLPS‐targeting small molecules.^32^, ^33^ Recent evidence shows that phase separation contributes to initiation and aggressiveness of human cancers.^29^ Herein, our results indicated that CNBP‐SMARCC2 condensates assembling through LLPS were essential for ribosome biogenesis in tumours. In humans, there are 15 SWI/SNF subunits that are assembled into canonical BRG1/BRM‐associated factor (BAF), polybromo‐associated BAF (PBAF) or non‐canonical BAF (ncBAF) complex.^34^ In these three complexes, homo‐ and heterodimers of SMARCC1 and SMARCC2 serve as initial core,^35^ while one of two mutually exclusive catalytic subunits, SMARCA4 or SMARCA2, exerts ATPase activity during nucleosome remodelling, gene transcription, DNA repair and replication.^34^, ^36^, ^37^ Mutations, translocations or deletions of SWI/SNF subunits are documented in 20% of malignancies, including germline mutations or copy number alterations of *SMARCA4*.^38^ As a key epigenetic regulator, *SMARCA4* affects the levels of *c‐Myc* or *cyclin D1* in acute leukaemia,^14^ lung cancer^15^ or colorectal carcinoma.^16^ In this study, we found that neither *CNBP*, *SMARCC2*, *SMARCC1* nor *SMARCA4* affected the levels of *c‐Myc* or *cyclin D1* in NB cells. In line with our findings, no changes in *c‐Myc* expression were noted in NB cells upon silencing of *SMARCA4*.^39^ Instead, we demonstrated that in a form of phase separation, CNBP interacted with and dissociated SMARCC2 from binding to target gene promoters, triggering the oncogenic roles of SMARCC1/SMARCA4 binary complex in NB progression.

Macrophages are mainly divided into M1 and M2 subtypes, while M2 macrophages exert oncogenic roles.^40^ Within microenvironment, tumour cells are able to recruit and differentiate M2‐type macrophages via secreting tumour necrosis factor.^41^ As a return, M2 macrophages facilitate tumour proliferation or invasion by activating Toll‐like receptor 4/signal transducer and activator of transcription 3.^42^ In this study, we found that NB cell‐derived EVs were able to mediate delivery of 18S rRNA into monocytic THP‐1 cells, resulting in differentiation of M2 macrophages. In addition, *CNBP* repressed the SMARCC2 activity to facilitate rRNA processing and ribosome biogenesis of tumour cells, leading to increase of M2 macrophage polarization. We further developed cell‐penetrating peptides blocking the phase separation and interaction of CNBP with SMARCC2, and achieved preferable therapeutic effects on NB progression, suggesting the essential roles of *CNBP*/*SMARCC2* axis in ribosomal biogenesis, M2 macrophage maturation and tumour progression.

## 4 CONCLUSIONS

In conclusion, we have shown that *CNBP* is up‐regulated and linked to poor outcome of NB. After nuclear entry mediated by KPNB1, CNBP inhibits the activity of SMARCC2/SMARCC1/SMARCA4 subunits via binding to SMARCC2, leading to alternative increase of SMARCC1/SMARCA4 binary complex‐facilitated gene expression essential for ribosome biogenesis and NB progression. In addition, *CNBP* is able to facilitate EV‐mediated delivery of 18S rRNA from tumour cells to monocytes, leading to M2 macrophage polarization. Blocking interaction between CNBP and SMARCC2 via cell‐penetrating peptide inhibits NB progression. This work broadens current literature regarding the functions of SWI/SNF subunits in controlling ribosome biogenesis, and suggests that *KPNB1*/*CNBP*/*SMARCC2* axis‐mediated ribosome biogenesis and M2 macrophage polarization is a valuable therapeutic target for tumours. Additional investigation is necessary to elucidate the therapeutic effects of targeting *CNBP*/*SMARCC2* in TH‐*MYCN* transgenic or immunocompetent mice, while quantitative proteomics are helpful in exploring additional CNBP protein partners in tumour progression.

## 5 METHODS

### 5.1 Cell lines and culture

The American Type Culture Collection (Rockville, MD, USA) and Pediatric Oncology Group Cell Bank (Lubbock, TX, USA) provided NB cells (SK‐N‐AS, SK‐N‐BE(2), SH‐SY5Y, BE(2)‐C, IMR‐32, SK‐N‐SH and NB‐1691), normal breast epithelial cell line (MCF 10A), HEK293T cells and monocyte cell line (THP‐1). After being revived from frozen aliquots, cell lines were subjected to short tandem repeat validation, and utilised for research with period less than 6 months. Lookout Mycoplasma PCR Detection Kit (Sigma, St. Louis, MO, USA) was used to check mycoplasma. Cell lines were cultivated using RPMI 1640 media supplemented with 10% foetal bovine serum (Merck Millipore, Burlington, MA, USA), and treated with IPZ (Sigma) or CX‐5461 (Selleck Chemicals, Houston, TX, USA).

### 5.2 Co‐culture of M2 macrophage

After treating with phorbol 12‐myristate 13‐acetate (10 ng mL^–1^) for 1 day, THP‐1 cells were subsequently incubated by interleukin 4 (25 ng mL^–1^) and interleukin 13 (25 ng mL^–1^) for 48 h. For co‐culture assay using six‐well plates with 1 µm pore inserts (Greiner‐Bio‐One, Frickenhausen, Germany), tumour cells (5 × 10^5^ cells/well) were grown at lower compartment, with THP‐1 cells or polaried M2 macrophages (5 × 10^5^ cells per well) seeded into upper compartment.

### 5.3 EV isolation and transfer assay

Conditional media of tumour cells were filtered using 0.22 µm filters, and treated by ultracentrifuging at 100 000 *g* for 90 min.^43^ The extracted EVs were measured by transmission electron microscopic observation, particle size assay, as well as western blotting assay utilising primary antibodies against CD9 (ab236630) and CD63 (ab134045, Abcam Inc., Waltham, MA, USA). Following a 30‐min labelling by fluorescent dye Dil (Sigma, 1:2000), purified EVs were suspended in 1000 µL of phosphate buffer saline (PBS) and administrated to culture medium.

### 5.4 Reverse transcription and real‐time polymerase chain reaction

The RNAiso Plus kit (Takara Bio Inc, Japan) was utilised to isolate total RNAs. Reverse transcription was undertaken with the PrimeScript RT Master Mix from Takara Bio Inc. The FastStart SYBR Green PCR Mix (Roche, Indianapolis, IN, USA) as well as primer sets (Table S7) were utilised for real‐time polymerase chain reaction (PCR), with transcript levels measured by the 2^−ΔΔCt^ method.^44^

### 5.5 Northern blot

TRIzol reagent (Sigma) was applied for preparing total RNAs. For the Northern blot, 20 µg of total RNAs were separated via formaldehyde‐agarose gel electrophoresis, shifted to the nitrocellulose membrane (Pall Corp., Port Washington, NY, USA), and incubated with biotin‐labelled 5′‐ITS‐1 or 18S rRNA probe (Table S7) for 16−18 h at 65°C. Blots were thoroughly washed, recognised by an anti‐horseradish peroxidase antibody, and detected on X‐ray films via chemiluminescence reactions (Roche).

### 5.6 Western blotting

The 1× cell lysis solution (Qiagen, Germantown, MD, USA) was applied in preparing the protein. Western blotting was carried out as documented,^45^, ^46^, ^47^, ^48^, ^49^, ^50^, ^51^, ^52^, ^53^ using antibodies against CNBP (ab48027), MYCN (ab16898), KPNB1 (ab2811), SMARCC2 (ab243634), SMARCC1 (ab305037), SMARCA4 (ab110641), BYSL (ab251811), NOP58 (ab155969), RRP9 (ab168845), c‐Myc (ab32072), cyclin D1 (ab16663), CD163 (ab182422), CD206 (ab64693), Flag‐tag (ab125243), β‐actin (ab8227), histone H3 (ab1791), glyceraldehyde‐3‐phosphate dehydrogenase (GAPDH; ab8245, Abcam Inc.), Myc‐tag (#2276), His‐tag (#9991) or GST‐tag (#2624, Cell Signaling Technology, Boston, MA, USA).

### 5.7 Ectopic expression or silencing of genes

By using PCR, *CNBP* cDNA (540 bp) and its truncated forms were prepared from NB specimens (Table S8), and inserted into the CV186 (Shanghai Genechem Co., Ltd., China), pET28a‐mCherry (Genprice Inc., San Jose, CA, USA), pmCherry‐N1 (Genprice Inc.) or pCMV‐3Tag‐1A (Addgene, Cambridge, MA, USA). Human *MYCN* expression vector was a kind gift from Dr. Arturo Sala (College of Health, Medicine and Life Sciences, Brunel University London).^54^ PCR primers (Table S8) were used to amplify human *SMARCC2* cDNA truncations (3645 bp) that were then subcloned into CV186 (Genechem Co., Ltd.), pCMV‐N‐MYC (Addgene) or pET28a‐EGFP (Genprice Inc.). Oligonucleotides specific for shRNAs against *CNBP*, *MYCN*, *KPNB1*, *SMARCC2*, *SMARCC1* or *SMARCA4* (Table S9) were ligated into GV298 vector (Genechem Co., Ltd). The dCas9‐BFP‐KRAB (Addgene) was used to produce single‐guide RNAs (sgRNAs; Table S9) against upstream or downstream regions of the transcription start sites for *CNBP*, *BYSL*, *NOP58* or *RRP9*. Puromycin (Invitrogen) screening was undertaken for establishment of stable cells.

### 5.8 Lentivirus preparation

The HEK293T cells were transfected by lentiviral vectors as well as packaging plasmids (psPAX2 and pMD2G) from Addgene. Sixty hours later, lentivirus was prepared via 0.45‐µm filters (Merck Millipore). Recombinant lentivirus was concentrated 100‐fold through ultracentrifugation (120 000 *g* for 2 h).

### 5.9 Transcriptome sequencing

By utilising the TRIzol reagent (Life Technologies, Inc.), total RNAs were isolated from tumour cells (1 × 10^6^). At Wuhan SeqHealth Technology Co., Ltd. (China), transcriptome sequencing and library preparation were undertaken on the Illumina HiSeq X Ten platform. Using the HTSeq v0.6.0 software, 100‐bp paired‐end raw reads were mapped, and fragments per kilobase of transcript per million fragments were examined. These data were submitted to Gene Expression Omnibus (GEO, accession code: GSE215748).

### 5.10 ChIP‐seq and sequential ChIP

ChIP was undertaken with Agarose ChIP Kit (Sigma),^45^, ^46^, ^47^, ^48^, ^49^, ^52^, ^55^ and antibodies against CNBP (sc‐515387, Santa Cruz Biotechnology, Inc., Dallas, TX, USA), SMARCC2 (#12760), SMARCC1 (#11956) or SMARCA4 (#49360, Cell Signaling Technology). Two confluent 55 cm^2^ dishes of cells (1 × 10^7^) were incubated with 0.75% formaldehyde for 10 min. After that, cells were treated by glycine (125 mmol L^−1^) for 5 min, rinsed twice with 10 mL cold PBS, scraped off dishes and transferred into 50 mL tube. The pellets were resuspended in 750 µL of ChIP lysis solution from Thermo Fisher Scientific, Inc. (Waltham, MA), while supernatant was carefully aspirated off. To produce 200−500 bp DNA fragments, lysates were sonicated on ice for 20 s. Then, 3 µg of specific antibodies or immunoglobulin G (IgG) was added to samples containing sheared chromatin for immunoprecipitation. Immuno‐complexes were rinsed by ChIP washing solution (Thermo Fisher Scientific, Inc.), and treated with elution solution (1 mol L^−1^ NaHCO_3_ and 1% sodium dodecyl sulphate [SDS]). To perform sequential ChIP, immuno‐complexes were eluted with Re‐ChIP elution solution (1% SDS and 15 mmol L^−1^ dithiothreitol), and then a second immunoprecipitation was conducted by utilising antibodies. The cross‐linking was reversed by 200 mmol L^−1^ NaCl and RNase A (Thermo Fisher Scientific, Inc.) at 65°C for 4 h, then bead pellets were recovered by a brief centrifugation at 600 *g* for 1 min. Samples were incubated with proteinase K (Thermo Fisher Scientific, Inc.) for 2 h, purified by using mini‐columns, treated by ethanol precipitation and resuspended in water. ChIP‐seq libraries were prepared by utilising common Nextera adapters (Illumina, Inc., San Diego, CA). On an Illumina Novaseq 6000 sequencer with PE150 model, 25 million reads per sample were sequenced at Wuhan SeqHealth Technology Co., Ltd. (China). The results were submitted to GEO database (accession code: GSE215795). The SYBR Green PCR Master Mix (Roche) and primer sets (Table S7) were used for real‐time qPCR assay.

### 5.11 Co‐immunoprecipitation and proteomic assay

Co‐immunoprecipitation was undertaken as documented,^46^, ^47^, ^48^, ^49^, ^52^, ^53^ using antibodies against CNBP (sc‐515387, Santa Cruz Biotechnology, Inc.), KPNB1 (ab2811), SMARCC2 (ab243634), Flag‐tag (ab125243, Abcam Inc.), Myc‐tag (#2276), His‐tag (#9991) or GST‐tag (#2624, Cell Signaling Technology). After being released from the bead‐bound complex, proteins were determined via western blotting or mass spectrometric assays (Wuhan SpecAlly Life Technology Co., Ltd., China).^46^, ^47^, ^52^, ^53^

### 5.12 Immunofluorescence observation

Cells were cultured on coverslips, which were treated by 10% goat serum for an hour at 37°C. Then, cells were treated by antibodies against KPNB1 (ab2811, Abcam Inc., dilution: 1:100), CNBP (sc‐515387, Santa Cruz Biotechnology, Inc., dilution: 1:100) or SMARCC2 (ab243634, Abcam Inc., dilution: 1:100) for 2 h. Coverslips were stained by 4′,6‐diamidino‐2‐phenylindole (300 nmol L^−1^) after incubating with Alexa Fluor 488 (ab150081) or Alexa Fluor 594 (ab150160, Abcam Inc.) goat anti‐rabbit IgG.

### 5.13 Bimolecular fluorescence complementation

The *CNBP* (540 bp) and *SMARCC2* (3645 bp) cDNA were, respectively, ligated into pBiFC‐VN173 or pBiFC‐VC155 (Addgene, Table S8). Following transfection by these vectors with Lipofectamine 3000 (Thermo Fisher Scientific, Inc.) for 1 day, tumour cells were grown for additional 10 h. A Nikon A1R‐SI confocal microscope was used to measure the fluorescence emission.^47^, ^53^

### 5.14 Inhibitory peptide synthesis

By using Peptiderive server,^18^ inhibitory peptides were designed to prevent interaction between CNBP and SMARCC2. Briefly, the Protein Data Bank (PDB) files of CNBP and SMARCC2 were generated by Phyre2 program,^56^ while their docking complex was analysed by ZDOCK program (https://zdock.umassmed.edu). The resulting PDB file was input into Peptiderive server (https://rosie.rosettacommons.org/peptiderive), while size of peptides was specified as 12 aa in length. The domains with highest docking score and corresponding inhibitory peptides were obtained. The cell‐penetrating peptides, YGRKKRRQRRR, were derived from transduction domain of Tat protein.^57^ Thus, N‐terminal biotin‐labelled cell‐penetrating peptides and C‐terminal fluorescein isothiocyanate were chemically conjugated to prepare inhibitory peptides (ChinaPeptides, Shanghai, China). Reversed‐phase high‐performance liquid chromatography assay was undertaken for evaluating purity of peptides (more than 95%).

### 5.15 Pull‐down using biotin‐labelled peptide

Using 1× cell lysis solution (Qiagen), cellular proteins were extracted. Then, biotin‐labelled peptide was added at 4°C for an overnight incubation. These peptide–protein complexes were incubated with streptavidin–agarose for 2 h at 4°C. After thoroughly washing the beads, pulled down proteins were obtained for western blotting.

### 5.16 Sucrose gradient sedimentation

Cells were treated by cycloheximide (100 g mL^−1^) for 10 min, while tumour tissues were pulverised under liquid nitrogen. Samples were reconstituted in hypotonic buffer (100 mg mL^−1^ cycloheximide, 1.5 mmol L^−1^ MgCl_2_, 10 mmol L^−1^ KCl, 0.5 mmol L^−1^ dithiothreitol, 10 mmol L^−1^ HEPES pH 7.9, 1× Protease Inhibitor Cocktail and 40 U mL^−1^ RNase inhibitor) for 15 min, and then shattered using a Dounce homogeniser (Kontes, Vineland, NJ, USA). Total proteins (1 mg) were added into 5%−50% gradient sucrose and spun at 250 000 *g* for 150 min, while the gradients were evaluated by a Piston Gradient Fractionator (BioComp Instruments, Canada).

### 5.17 Protein synthesis assay

Tumour cells underwent puromycin (10 µg mL^–1^) treatment for 15 min. Puromycylated peptides were measured via western blotting with antibody against puromycin (MABE343; Merck Millipore; 1:8000). To visualise protein synthesis, OP‐puro labelling assay was undertaken with EZClick Global Protein Synthesis Assay Kit (BioVision, Milpitas, CA, USA). Quantification of newly synthesised OP‐puro‐labelled peptides was performed by analysing percentage of positive cells.

### 5.18 In vitro phase separation assay

For preparing recombinant proteins, His‐tagged *CNBP* or *SMARCC2* construct was transferred into *Escherichia coli* BL21 strain (Thermo Fisher Scientific, Inc.), while proteins were purified by His‐tag Protein Purification Kit (Thermo Fisher Scientific, Inc.). The recombinant proteins were detected by SDS‐PAGE and Coomassie blue staining, while their purity was determined by densitometry with ImageJ (https://imagej.nih.gov/ij) program. In brief, after background correction, the density of recombinant protein band was divided by that of whole lane, which was multiplied by 100%. On glass‐bottomed dishes, a phase separation test was performed. In droplet formation buffer (1 mmol L^−1^ dithiothreitol, 10% glycerol, 50 mmol L^−1^ Tris–HCl, pH 7.5), 40 µmol L^−1^ of proteins were incubated with solutions containing crowding agent (10% polyethylene glycol‐8000) and observed with a Nikon A1R‐SI confocal microscope outfitted with oil‐immersion lenses.

### 5.19 In vivo phase separation assay

Tumour cells stably expressing mCherry‐tagged protein or endogenous protein were grown on glass‐bottomed dishes, washed twice with PBS and labelled with Hoechst 33342 (Thermo Fisher Scientific, Inc.) for 10 min. Cells were examined using a Nikon A1R‐SI confocal microscope following two PBS washes. The phase separation puncta were defined as visible ones with diameter more than 0.5 µm.

### 5.20 FRAP assay

During in vitro tests, droplets were photobleached with 50% laser power (488 and 561 nm pulses) for 10 s, while time‐series photos were taken by using a Nikon A1R‐SI confocal microscope outfitted with oil‐immersion objectives. In a live‐cell imaging chamber, the FRAP test was carried out for in vivo research. Laser pulses at 488 and 561 nm were used to bleach the droplets for 5 s at 50% power, and photobleaching recovery was observed for 1 min. Utilising the FIJI/ImageJ program, fluorescence intensities were normalised to those prior to photobleaching.

### 5.21 Cellular apoptosis and cell cycle assays

For apoptosis detection, tumour cells (5 × 10^5^) were treated with 5 µL of propidium iodide and Annexin V‐FITC (Sigma) in dark for 15 min, and assessed at 488 nm wavelength on a flow cytometer (BD Biosciences, San Jose, CA, USA). In cell cycle assay, tumour cells were treated with 70% ice‐cold ethanol and RNase A (2 mg mL^–1^; Sigma). Then, cells were treated by propidium iodide (20 mg mL^–1^; Sigma) for 20 min. A flow cytometer (BD Biosciences) was used to measure cell cycle phases.

### 5.22 Cellular viability assay

In each well of 96‐well plates, 3 × 10^3^ of tumour cells were treated by 5 mg mL^–1^ of 2‐(4,5‐dimethyltriazol‐2‐yl)−2,5‐diphenyl tetrazolium bromide (MTT, Sigma). After 4 h of incubation at 37°C, cell supernatants were removed. MTT crystals were dissolved in 150 µL of dimethylsulphoxide (Sigma), while absorbance was measured at 570 nm.^53^

### 5.23 Anchorage‐independent growth assay

On 6‐well plates coated with 0.1% Noble agar, tumour cells (5 × 10^3^ each well) were mixed with 0.05% Nobel agar (Thermo Fisher Scientific, Inc.) and cultured for 21 days. Under an Olympus BX43 microscope, the colonies were quantified after staining by 0.5% crystal violet.^45^, ^46^, ^47^, ^48^, ^49^, ^50^, ^52^

### 5.24 Cellular invasion assay

Tumour cells (1 × 10^5^ cells) were grown in serum‐free media, and introduced to upper chambers of six‐well plates coated with Matrigel matrix (BD Biosciences). Twenty‐four hours later, invaded cells were stained by 0.1% crystal violet and quantified.^45^, ^46^, ^47^, ^48^, ^49^, ^50^, ^52^, ^58^

### 5.25 Nude mice assays

Nude mice studies were undertaken with Guidelines for the Care and Use of Laboratory Animals set forth by National Institutes of Health, and were approved by Experimental Animal Ethics, Huazhong University of Science and Technology (permission number: 2019‐3183). To conduct investigation on subcutaneous xenograft tumours or experimental metastasis, randomised male BALB/c nude mice of 4 weeks at age (*n* = 5 each group) received injection of tumour cells (1 × 10^6^ or 0.4 × 10^6^ each mouse).^45^, ^46^, ^47^, ^52^, ^53^ In therapeutic investigations, tumour cells (1 × 10^6^ or 0.4 × 10^6^) were administrated into mice's dorsal flanks or tail vein. Two weeks later, inhibitory peptides were administrated via tail vein. The mice were sacrificed when presence of 20% weight loss was recognised as humane endpoint.

### 5.26 Human tissue sample

The investigation using human specimens was authorised by Institutional Review Board of Union Hospital, Tongji Medical College, Huazhong University of Science and Technology (permission number: 2017‐S242), and were in compliance with Declaration of Helsinki's recommendations. All patients' legal guardians presented their written agreement. None of the cases received any kind of preoperative care, including chemotherapy. From aborted pregnancies, normal dorsal root ganglia were obtained. During surgery, tumour specimens were removed and stored at −80°C.

### 5.27 Immunohistochemical staining

Immunohistochemistry was undertaken with antibodies against CNBP (sc‐515387, dilution: 1:200), Ki‐67 (sc‐23900, Santa Cruz Biotechnology, Inc., dilution: 1:200), CD31 (Arigo, ARG52748, Arigo, Hsinchu City, Taiwan, 1:100 dilution), CD3 (ab135372, dilution: 1:200) or F4/80 (ab16911, Abcam Inc., dilution: 1:200). Using an Olympus BX43 microscope (Tokyo, Japan), 10 distinct fields of high power (400×) were viewed for each specimen, while positive cell percentage was evaluated via Image‐Pro Plus 6.0 (Media Cybernetics, Rockville, MD, USA).^45^, ^47^, ^49^

### 5.28 Argyrophilic nucleolar organiser region staining

NORs were stained as previously explained.^59^ After fixation and rehydration, specimens were incubated with argyrophilic nucleolar organiser region staining solution for 30 min, treated by 5% sodium thiosulphate for 2–5 min, rinsed once again, and observed under a bright field microscopy.

### 5.29 Statistical analysis

For statistical analysis, GraphPad Prism 9 and SPSS 20.0 programs were used, which were obtained from GraphPad Software (San Diego, CA, USA) and SPSS Inc. (Chicago, IL, USA), respectively. The data were presented by mean and standard error of the mean. The cutoffs of gene expression were determined by medium or average levels. The difference was compared by using Pearson chi‐square test, one‐way analysis of variance or two‐sided unpaired Student's *t* test. Fisher's exact test and Pearson's product–moment correlation assay were used for statistical analysis of overlap or expression correlation. The hazard ratio and difference in survival were evaluated by log‐rank test and Cox regression model. A two‐sided statistical analysis was used, with *p*‐values less than .05 considered statistically significant.

## CONFLICT OF INTEREST STATEMENT

The authors declare that they have no competing interests.

## CONSENT FOR PUBLICATION

Written informed consent was obtained from a leagally authorized representative of all patients.

## Supporting information

Supporting Information

Supporting Information

Supporting Information

Supporting Information

Supporting Information

Supporting Information
