# Supplementary material for: Therapeutic targeting of CNBP phase separation inhibits ribosome biogenesis and neuroblastoma progression via modulating SWI/SNF complex activity
Source: Clin Transl Med. 2023 Apr 26;13(4):e1235. doi: 10.1002/ctm2.1235 (PMC10131295; doi:10.1002/ctm2.1235)
Supplement: Supplementary file 2 — Supporting Information [file CTM2-13-e1235-s005.doc]

**Table S1 Identification of transcriptional regulator essential for NB progression**

| **Transcriptional regulators essential for NB progression** | | | | |  | **Survival significance of 18 transcriptional regulators** | | | | |
| --- | --- | --- | --- | --- | --- | --- | --- | --- | --- | --- |
| **TARGET** |  | **GSE62564** | | |  | **TARGET** | **GSE62564** | **gencode19** | **GSE3446** | **GSE16476** |
| BRCA1 |  | AATF | MESP2 | TEAD4 |  | BRCA1 | BRCA1 | CNBP | CNBP | BRCA1 |
| CEBPG |  | ABT1 | MGA | TFAM |  | CNBP | CEBPG | E2F1 | ELK1 | CNBP |
| CNBP |  | ACTL6A | MTA2 | TFAP2D |  | E2F1 | CNBP | E2F3 | TCF3 | E2F1 |
| E2F1 |  | AIP | MXD3 | TFAP4 |  | E2F3 | E2F1 | E2F8 | UBN1 | E2F3 |
| E2F3 |  | ALX1 | MYBL1 | TFB2M |  | E2F8 | E2F3 | ELK1 |  | E2F8 |
| E2F8 |  | APEX1 | MYBL2 | TGIF2 |  | ELK1 | E2F8 | MYBL2 |  | GATAD2A |
| ELK1 |  | ASCL1 | MYCN | THAP11 |  | GATAD2A | ELK1 | NPM1 |  | HMGB2 |
| GATAD2A |  | ATF4 | MYNN | TLX2 |  | HMGB2 | GATAD2A | RCOR2 |  | MYBL2 |
| HMGB2 |  | ATF5 | MYPOP | TONSL |  | MYBL2 | HMGB2 | SCML1 |  | NPM1 |
| MYBL2 |  | BARX1 | NEUROD1 | TP53 |  | NPM1 | MYBL2 | UHRF1 |  | RCOR2 |
| NPM1 |  | BRCA1 | NFATC3 | TRIM28 |  | RCOR2 | NPM1 |  |  | TCF3 |
| RCOR2 |  | C1QBP | NFIB | TRIP13 |  | TCF3 | RCOR2 |  |  | TONSL |
| SCML1 |  | CCNE1 | NFXL1 | TTF1 |  | TONSL | SCML1 |  |  | UHRF1 |
| TCF3 |  | CCRN4L | NFYA | TWIST1 |  | UHRF1 | TCF3 |  |  | ZNF367 |
| TONSL |  | CDK7 | NFYB | UBN1 |  |  | TONSL |  |  |  |
| UBN1 |  | CEBPG | NHLH2 | UHRF1 |  |  | UBN1 |  |  |  |
| UHRF1 |  | CEBPZ | NKRF | USP16 |  |  | UHRF1 |  |  |  |
| ZNF367 |  | CHCHD3 | NKX6-2 | VAX2 |  |  | ZNF367 |  |  |  |
|  |  | CITED1 | NME2 | VSX1 |  |  |  |  |  |  |
|  |  | CNBP | NPM1 | WDR77 |  |  |  |  |  |  |
|  |  | CNOT7 | NR6A1 | WT1 |  |  |  |  |  |  |
|  |  | CREB3L4 | NUCKS1 | WWC1 |  |  |  |  |  |  |
|  |  | CTBP1 | PA2G4 | YEATS4 |  |  |  |  |  |  |
|  |  | CTCFL | PARP1 | YY1 |  |  |  |  |  |  |
|  |  | DAXX | PCGF6 | ZBED5 |  |  |  |  |  |  |
|  |  | DBP | PFDN5 | ZBED6 |  |  |  |  |  |  |
|  |  | DDN | PHB | ZBTB25 |  |  |  |  |  |  |
|  |  | DDX1 | PHF21B | ZFP82 |  |  |  |  |  |  |
|  |  | DDX54 | PHF5A | ZIC2 |  |  |  |  |  |  |
|  |  | DLX3 | PHF6 | ZNF121 |  |  |  |  |  |  |
|  |  | DLX5 | PIAS2 | ZNF132 |  |  |  |  |  |  |
|  |  | DLX6 | PIR | ZNF135 |  |  |  |  |  |  |
|  |  | DUX4 | PITX1 | ZNF138 |  |  |  |  |  |  |
|  |  | E2F1 | PKN1 | ZNF146 |  |  |  |  |  |  |
|  |  | E2F3 | POU5F1B | ZNF157 |  |  |  |  |  |  |
|  |  | E2F6 | POU6F2 | ZNF16 |  |  |  |  |  |  |
|  |  | E2F8 | PQBP1 | ZNF174 |  |  |  |  |  |  |
|  |  | EBF3 | PRDM5 | ZNF219 |  |  |  |  |  |  |
|  |  | ECD | PRKRIR | ZNF227 |  |  |  |  |  |  |
|  |  | EDF1 | PRMT5 | ZNF239 |  |  |  |  |  |  |
|  |  | ELK1 | PRPF6 | ZNF24 |  |  |  |  |  |  |
|  |  | EN1 | PRRX2 | ZNF256 |  |  |  |  |  |  |
|  |  | ENO1 | PTTG1 | ZNF26 |  |  |  |  |  |  |
|  |  | ESRRA | RAN | ZNF263 |  |  |  |  |  |  |
|  |  | ESRRB | RAX2 | ZNF302 |  |  |  |  |  |  |
|  |  | ETV4 | RBM14 | ZNF367 |  |  |  |  |  |  |
|  |  | FERD3L | RCOR2 | ZNF420 |  |  |  |  |  |  |
|  |  | FEV | REXO4 | ZNF45 |  |  |  |  |  |  |
|  |  | FOXI3 | RFXANK | ZNF496 |  |  |  |  |  |  |
|  |  | FOXK2 | RLIM | ZNF543 |  |  |  |  |  |  |
|  |  | FOXM1 | RNF4 | ZNF567 |  |  |  |  |  |  |
|  |  | GATAD2A | RRN3 | ZNF572 |  |  |  |  |  |  |
|  |  | GMEB2 | SAP30 | ZNF573 |  |  |  |  |  |  |
|  |  | GMNN | SCAND1 | ZNF578 |  |  |  |  |  |  |
|  |  | GSC | SCML1 | ZNF585A |  |  |  |  |  |  |
|  |  | GTF2A2 | SCML2 | ZNF595 |  |  |  |  |  |  |
|  |  | GTF2F1 | SCRT2 | ZNF605 |  |  |  |  |  |  |
|  |  | GTF3C3 | SETD3 | ZNF607 |  |  |  |  |  |  |
|  |  | HES6 | SF1 | ZNF621 |  |  |  |  |  |  |
|  |  | HIF3A | SFR1 | ZNF630 |  |  |  |  |  |  |
|  |  | HMBOX1 | SIAH2 | ZNF639 |  |  |  |  |  |  |
|  |  | HMGA1 | SIX2 | ZNF668 |  |  |  |  |  |  |
|  |  | HMGB2 | SLC30A9 | ZNF717 |  |  |  |  |  |  |
|  |  | HNRNPAB | SMARCD2 | ZNF750 |  |  |  |  |  |  |
|  |  | HNRNPK | SNW1 | ZNF808 |  |  |  |  |  |  |
|  |  | HOXD10 | SOHLH2 | ZNF829 |  |  |  |  |  |  |
|  |  | HOXD13 | SOX12 | ZNF880 |  |  |  |  |  |  |
|  |  | HOXD8 | SPZ1 | ZNF90 |  |  |  |  |  |  |
|  |  | HOXD9 | SRF | ZNF93 |  |  |  |  |  |  |
|  |  | KIAA1958 | SRSF2 |  |  |  |  |  |  |  |
|  |  | KLF1 | SS18L2 |  |  |  |  |  |  |  |
|  |  | KLF15 | TAF1 |  |  |  |  |  |  |  |
|  |  | KLF16 | TAF10 |  |  |  |  |  |  |  |
|  |  | LHX3 | TAF6L |  |  |  |  |  |  |  |
|  |  | MED12 | TAF7 |  |  |  |  |  |  |  |
|  |  | MED26 | TAF7L |  |  |  |  |  |  |  |
|  |  | MED27 | TBL1X |  |  |  |  |  |  |  |
|  |  | MED6 | TBX4 |  |  |  |  |  |  |  |
|  |  | MED7 | TBX6 |  |  |  |  |  |  |  |
|  |  | MESP1 | TCF3 |  |  |  |  |  |  |  |
