# Supplementary material for: Therapeutic targeting of CNBP phase separation inhibits ribosome biogenesis and neuroblastoma progression via modulating SWI/SNF complex activity
Source: Clin Transl Med. 2023 Apr 26;13(4):e1235. doi: 10.1002/ctm2.1235 (PMC10131295; doi:10.1002/ctm2.1235)
Supplement: Supplementary file 4 — Supporting Information [file CTM2-13-e1235-s004.doc]

**Table S3 Univariate and multivariate analysis of prognostic factors in 498 NB**

**patients (GSE62564)**

| **Variables** | ***n*** | **Univariate analysis** | | **Multivariate analysis** | | |
| --- | --- | --- | --- | --- | --- | --- |
|  |  | Mean ± SEM (months) | *P*-value | Hazard ratio | 95% CI | *P*-value |
| Age |  |  |  |  |  |  |
| ≤18 months | 299 | 202.6 ± 2.9 |  |  |  |  |
| >18 months | 199 | 125.1 ± 7.6 | <0.001 | 2.416 | 1.285-4.540 | 0.006 |
|  |  |  |  |  |  |  |
| Gender |  |  |  |  |  |  |
| Female | 211 | 150.9 ± 5.5 |  |  |  |  |
| Male | 287 | 178.3 ± 5.0 | 0.252 | 0.727 | 0.488-1.083 | 0.117 |
|  |  |  |  |  |  |  |
| *MYCN* amplification | |  |  |  |  |  |
| No | 406 | 191.0 ± 3.7 |  |  |  |  |
| Yes | 92 | 63.4 ± 6.5 | <0.001 | 3.410 | 2.210-5.260 | <0.001 |
|  |  |  |  |  |  |  |
| INSS stages | |  |  |  |  |  |
| Stage 1 | 121 | 193.9 ± 1.5 |  |  |  |  |
| Stage 2 | 78 | 161.1 ± 3.8 |  | 0.151 | 0.016-1.397 | 0.096 |
| Stage 3 | 63 | 170.4 ± 10.7 | <0.001 | 0.509 | 0.121-2.133 | 0.355 |
| Stage 4 | 183 | 117.4 ± 8.3 |  | 1.061 | 0.322-3.492 | 0.922 |
| Stage 4S | 53 | 156.4 ± 5.9 |  | 1.090 | 0.338-3.519 | 0.885 |
|  |  |  |  |  |  |  |
| High risk | |  |  |  |  |  |
| No | 322 | 207.6 ± 2.3 |  |  |  |  |
| Yes | 176 | 95.9 ± 9.2 | <0.001 | 5.347 | 2.068-13.823 | 0.001 |
|  |  |  |  |  |  |  |
| CNBP expression | |  |  |  |  |  |
| Low | 249 | 202.7 ± 3.3 |  |  |  |  |
| High | 249 | 139.7 ± 6.9 | <0.001 | 1.808 | 1.007-3.244 | 0.047 |

INSS, international neuroblastoma staging system; CNBP, cellular nucleic acid binding protein; Log-rank test and Cox regression model were applied for univariate and multivariate analysis. *n*, number of patients; SEM, standard error of the mean; 95% CI, 95% confidence interval.

***Table S4 Mass spectrometry analysis of proteins pulled down by CNBP antibody***

| **Mock** | |  | ***CNBP*** | | | | | |
| --- | --- | --- | --- | --- | --- | --- | --- | --- |
| ANPEP | PTMA |  | ABLIM1 | DDX6 | INA | NUDT21 | SIPA1L1 | XPNPEP3 |
| APC | RAI1 |  | ACT | DEF6 | INPP5K | PACSIN3 | SKP1 | XRCC5 |
| ARF4 | RBM15 |  | ACTA1 | DHX15 | IQGAP1 | PALMD | SLC25A13 | YWHAG |
| ARPC4 | RBM3 |  | ACTA2 | DHX36 | IRS2 | PDLIM7 | SLC25A3 | YWHAQ |
| BST2 | RCN2 |  | ACTG1 | DHX57 | ITPR3 | PELO | SLC25A5 | YWHAZ |
| C1orf57 | RPAP3 |  | ACTN1 | DNCL1 | JUP | PHF5A | SLC25A6 | ZNF185 |
| CAAP1 | RPLP1 |  | ACTN4 | DNTTIP1 | KIAA1671 | PKP3 | SLC2A1 |  |
| CACNA2D1 | RPN2 |  | ACTR2 | DSP | KIF14 | PKP4 | SMARCA4 |  |
| CAD | S100P |  | ACTR3 | DST | KIF2B | PLEC | SMARCC1 |  |
| CALU | SCRIB |  | ADAR | DVL3 | KPNB1 | PLEC1 | SMARCC2 |  |
| CAMK2G | SERPINH1 |  | AFAP1 | DYNC2H1 | LACTB | PLEKHA6 | SMC3 |  |
| CAV2 | SLC2A1 |  | AHNAK | DYR | LARP1 | PLEKHA7 | SNRNP200 |  |
| CD44 | SMARCC2 |  | AIF1L | EEF1A2 | LENG9 | PLEKHG3 | SNRPF |  |
| CD55 | SMC3 |  | AKAP2 | EEF2 | LGALS1 | POLR2E | SORBS2 |  |
| CPNE4 | STOM |  | ALPI | EFHD1 | LGALS3 | PPL | SPATA5L1 |  |
| CSNK1D | SYNM |  | ALPL | EGFR | LGALS8 | PPP1CA | SPECC1 |  |
| CYSRT1 | TCEB2 |  | ALPP | EIF2S2 | LIMA1 | PPP1CB | SPTAN1 |  |
| DARS | TJP2 |  | ANKHD1 | EIF2S3L | LIMCH1 | PPP1CC | SPTBN1 |  |
| DNTTIP1 | TOR4A |  | ANPEP | EIF3H | LMNA | PPP1R12A | SPTBN2 |  |
| DST | TRIM21 |  | ANXA2 | EIF4ENIF1 | LMNB1 | PPP1R18 | SRP9 |  |
| DVL3 | UNC45A |  | APC | EMD | LMNB2 | PPP1R9A | SSBP1 |  |
| DYR | UQCRC2 |  | ARF4 | EPB41L1 | LMO7 | PPP3CA | SSFA2 |  |
| EGFR | USP6NL |  | ARL6IP4 | EPPK1 | LONP1 | PRC1 | SSRP1 |  |
| EIF3H | XPNPEP3 |  | ARPC2 | EVPL | LRCH3 | PRDX6 | STOM |  |
| EIF4ENIF1 |  |  | ARPC4 | EWSR1 | LRRC59 | PRKDC | STRBP |  |
| EPB41L1 |  |  | ASCC3 | FAM64A | LRRFIP2 | PROSER2 | SVIL |  |
| ESPN |  |  | ATP2A2 | FAM83B | LUZP1 | PRPF19 | SYNM |  |
| EVPL |  |  | ATP6V1D | FAM83G | MACF1 | PRPF8 | TBL2 |  |
| FAM64A |  |  | AURKB | FARP1 | MCM7 | PTMA | TCEB2 |  |
| FARP1 |  |  | BANF1 | FLII | MICAL3 | PTRF | TECR |  |
| FLOT1 |  |  | BASP1 | FLNA | MISP | RAE1 | THAP11 |  |
| GLB1 |  |  | BCLAF1 | FLNB | MPRIP | RAI1 | THRAP3 |  |
| GNB1 |  |  | BST2 | FLOT1 | MRPL14 | RAI14 | TJP1 |  |
| GNG12 |  |  | CAAP1 | FN1 | MRPS21 | RBM15 | TJP2 |  |
| GPRC5A |  |  | CACNA2D1 | FOLR1 | MSH6 | RBM3 | TMOD1 |  |
| GRID2 |  |  | CAD | GAN | MTCL1 | RCN2 | TMOD3 |  |
| HEL-S-128m |  |  | CALM3 | GLB1 | MTDH | RFC4 | TMPO |  |
| HEL-S-77p |  |  | CALU | GNAI2 | MUC1 | RGPD3 | TNKS1BP1 |  |
| HMGN1 |  |  | CAMK2G | GNAI3 | MUC13 | RNF40 | TOP2A |  |
| HSGT1 |  |  | CAPZA2 | GNAS | MYBBP1A | RPAP3 | TOR4A |  |
| IGKV1-8 |  |  | CAPZB | GNB1 | MYCBP | RPL10A | TPM1 |  |
| IGLV3-21 |  |  | CAV2 | GNB2 | MYH1 | RPL21 | TPM2 |  |
| INPP5K |  |  | CCAR1 | GNG12 | MYH2 | RPL34 | TPM3 |  |
| IRS2 |  |  | CD44 | GPRC5A | MYH4 | RPL35A | TPM4 |  |
| KPNB1 |  |  | CD55 | GRID2 | MYH7 | RPL39 | TPRN |  |
| LACTB |  |  | CD59 | GSN | MYH9 | RPL5 | TRIM21 |  |
| LGALS8 |  |  | CDC42EP1 | H2AFY | MYL12A | RPL6 | TRIM27 |  |
| LONP1 |  |  | CDK1 | HIST1H2AH | MYL6 | RPL7 | TRIOBP |  |
| LRCH3 |  |  | CKAP4 | HIST1H2BJ | MYL9 | RPLP0 | TST |  |
| LRRC59 |  |  | CLPX | HIST2H2AB | MYLK | RPLP1 | TUBA1B |  |
| MACF1 |  |  | COBL | HIST2H3A | MYO1B | RPLP2 | TUBA1C |  |
| MRPS21 |  |  | CORO1C | HMGA1 | MYO1C | RPN1 | TUBB |  |
| MSH6 |  |  | CORO2A | HMGN1 | MYO1E | RPN2 | TUBB2B |  |
| MUC13 |  |  | CPNE4 | HRNR | MYO5A | RPS10 | TUBB2C |  |
| MYO5A |  |  | CPSF7 | HSGT1 | MYO5C | RPS5 | TUBB6 |  |
| MYO5C |  |  | CSNK1A1 | HSPA5 | MYO6 | S100A10 | TUBB8 |  |
| NKD2 |  |  | CSNK1D | HSPA8 | NAP1L1 | S100P | TUFM |  |
| NT5E |  |  | CTSA | HSPA9 | NCAM2 | SAFB2 | UACA |  |
| NUP133 |  |  | CYSRT1 | HSPB1 | NES | SCIN | UNC45A |  |
| PALMD |  |  | CYTSA | IARS | NEXN | SCRIB | UQCRC2 |  |
| PLEC1 |  |  | DAPK3 | IGHG1 | NKD2 | SERPINH1 | USMG5 |  |
| PLEKHA7 |  |  | DARS | IGKV1-8 | NKRF | SF3B1 | USP6NL |  |
| POLR2E |  |  | DBN1 | IGLV3-12 | NT5E | SF3B6 | VASP |  |
| PPP3CA |  |  | DDX48 | IMPDH2 | NTPCR | SIPA1 | VDAC2 |  |
