# Supplementary material for: Therapeutic targeting of CNBP phase separation inhibits ribosome biogenesis and neuroblastoma progression via modulating SWI/SNF complex activity
Source: Clin Transl Med. 2023 Apr 26;13(4):e1235. doi: 10.1002/ctm2.1235 (PMC10131295; doi:10.1002/ctm2.1235)
Supplement: Supplementary file 6 — Supporting Information [file CTM2-13-e1235-s001.doc]

**Table S6 GO pathway analysis of *CNBP*/*SMARCC2* target genes**

| **Description** | **-Log (*P*-value)** |
| --- | --- |
| phosphatidylinositol-mediated signaling | 3.639789 |
| myeloid cell differentiation | 3.666127 |
| ribosome biogenesis | 3.689163 |
| cellular component biogenesis | 3.711890 |
| cell migration | 3.991621 |
| cell projection organization | 4.091020 |
| microtubule cytoskeleton organization | 4.250151 |
| cell division | 4.458261 |
| rRNA processing | 4.490090 |
| response to insulin | 4.633774 |

CNBP, CCHC-type zinc finger nucleic acid binding protein; SMARCC2, SWI/SNF related matrix associated actin dependent regulator of chromatin subfamily c member 2.

**Table S7 Primer sets used for RT-PCR, qPCR, ChIP, and probe**

| **Primer set** | **Primers** | **Sequence** | **Product size (bp)** | **Application** |
| --- | --- | --- | --- | --- |
| CNBP | Forward | 5’-GCTACAACTGTGGCAAACC-3' | 213 | RT-PCR |
|  | Reverse | 5’-GTGCAAGGTGCCCTGACT-3' |  |  |
| BYSL | Forward | 5’-CAGCAAGCACGGCAGCAACA-3’ | 208 | RT-PCR |
|  | Reverse | 5’-CCTCAGGGTCCACAACCACC-3' |  |  |
| NOP58 | Forward | 5’-ACAGAAAGTTGGCGATAGGAAG-3' | 189 | RT-PCR |
|  | Reverse | 5’-AGCTGGGTTCGATATTCAGAGA-3' |  |  |
| RRP9 | Forward | 5’-CTCCTCCGACGGCAAGTACC-3' | 129 | RT-PCR |
|  | Reverse | 5’-CGGAATGCCAGACCCGACAC-3' |  |  |
| IL10 | Forward | 5’-AAGCCCGTATGCTTGTAAAG-3' | 267 | RT-PCR |
|  | Reverse | 5’-ACATGGAAGGAGATGACCCT-3' |  |  |
| TGFB1 | Forward | 5’-GCAACAATTCCTGGCGATAC-3' | 136 | RT-PCR |
|  | Reverse | 5’-SCTAAGGCGAAAGCCCTCAAT-3' |  |  |
| β-actin | Forward | 5'-TGCCCATCTACGAGGGGTATG-3' | 156 | RT-PCR, qPCR |
|  | Reverse | 5'-TCTCCTTAATGTCACGCACGATTT-3' |  |  |
| GAPDH | Forward | 5'-AGAAGGCTGGGGCTCATTTG-3' | 258 | qPCR |
|  | Reverse | 5'-AGGGGCCATCCACAGTCTTC-3' |  |  |
| BYSL  (-82/+118) | Forward | 5'-GCTGATGGCGCTTCTGGCCTCC-3' | 201 | ChIP |
|  | Reverse | 5'-GGGGCGCATGTTTTTCCTGACC-3' |  |  |
| NOP58  (-1010/-725) | Forward | 5'-AACATCGTGAAACCCTGTCTCT-3' | 286 | ChIP |
|  | Reverse | 5'-CTCTTGACCTCGTGATACGCCC-3' |  |  |
| RRP9  (-233/-33) | Forward | 5'-GCTGATGGCGCTTCTGGCCTCC-3' | 201 | ChIP |
|  | Reverse | 5'-GGGGCGCATGTTTTTCCTGACC-3' |  |  |
| T7+5’-ITS1 | Forward | 5’-TAATACGACTCACTATAGGGCCTCGCCCTCCGGGCTCCGTTAATGATC-3’ |  | Probe |
|  | Reverse | 5’-GATCATTAACGGAGCCCGGAGGGCGAGGCCCTATAGTGAGTCGTATTA-3’ |  |  |
| T7+18S | Forward | 5’-TAATACGACTCACTATAGGGGCATGGCTTAATCTTTGAGACAAGCATAT-3’ |  | Probe |
|  | Reverse | 5’-ATATGCTTGTCTCAAAGATTAAGCCATGCCCCTATAGTGAGTCGTATTA-3’ |  |  |

CNBP, CCHC-type zinc finger nucleic acid binding protein; BYSL, bystin like; NOP58, NOP58 ribonucleoprotein; RRP9, ribosomal RNA processing 9; IL10, interleukin 10; TGFB1, transforming growth factor beta 1; GAPDH, glyceraldehyde 3-phosphate dehydrogenase; ChIP, chromatin immunoprecipitation.

**Table S8 Primer sets used for constructs**

| **Primer set** | **Primers** | **Sequence** |
| --- | --- | --- |
|  |  |  |
| CV186-CNBP | Forward | 5'-CGCGGATCCATGAGCAGCAATGAGTG-3' |
|  | Reverse | 5'-GCGCACCGGTTTAGGCTGTAGCCTCA-3' |
| pCMV-3Tag-1A-CNBP | Forward | 5'-CGCGGATCCATGAGCAGCAATGAGTGCTT-3' |
|  | Reverse | 5'-CCGCTCGAGTTAGGCTGTAGCCTCAATTG-3' |
| pCMV-3Tag-1A-CNBP-ΔRGG | Forward | 5'-ATGTCCTACTTTCCAGTTTGTTTCCTCGTC-3' |
|  | Reverse | 5'-CAAACTGGAAAGTAGGACATTCCCGGGCCC-3' |
| pCMV-3Tag-1A-CNBP-ΔZn1-RGG | Forward | 5'-CGCGGATCCCCAGACATTTGTTATCGCTG-3' |
|  | Reverse | 5'-CCGCTCGAGTTAGGCTGTAGCCTCAATTG-3' |
| pCMV-3Tag-1A-CNBP-ΔZn5-7 | Forward | 5'-CGCGGATCCATGAGCAGCAATGAGTGCTT-3' |
|  | Reverse | 5'-CCGCTCGAGTGCATGGTCGCAGTCACGAG-3' |
| pGEX-6P-1-CNBP | Forward | 5'-CGCGGATCCATGAGCAGCAATGAGTGCTT-3' |
|  | Reverse | 5'-CCGCTCGAGTTAGGCTGTAGCCTCAATTG-3' |
| pGEX-6P-1-CNBP-ΔRGG | Forward | 5'-ATGTCCTACTTTCCAGTTTGTTTCCTCGTC-3' |
|  | Reverse | 5'-CAAACTGGAAAGTAGGACATTCCCGGGCCC-3' |
| pGEX-6P-1-CNBP-ΔZn1-RGG | Forward | 5'-CGCGGATCCCCAGACATTTGTTATCGCTG-3' |
|  | Reverse | 5'-CCGCTCGAGTTAGGCTGTAGCCTCAATTG-3' |
| pGEX-6P-1-CNBP-ΔZn5-7 | Forward | 5'-CGCGGATCCATGAGCAGCAATGAGTGCTT-3' |
|  | Reverse | 5'-CCGCTCGAGTGCATGGTCGCAGTCACGAG-3' |
| CV186-KPNB1 | Forward | 5'-CGCGGATCCATGGAGCTGATCACCA-3' |
|  | Reverse | 5'-GCGCACCGGTTCAAGCTTGGTTCTT-3' |
| pMAL-c4X-KPNB1 | Forward | 5'-CGCGGATCCATGGAGCTGATCACCATTCTCGAG-3' |
|  | Reverse | 5'-TGCTCTAGATCAAGCTTGGTTCTTCAGTTTCCT-3' |
| pCMV-N-Myc-KPNB1 | Forward | 5'-CGCGGATCCATGGAGCTGATCACCATTCTCGAG-3' |
|  | Reverse | 5'-TGCTCTAGATCAAGCTTGGTTCTTCAGTTTCCT -3' |
| CV186-SMARCC2 | Forward | 5’-CTTGGGCTGCAGGTCGACTCTAGAGGATCCATGGCGGT-3' |
|  | Reverse | 5’-GTCATCGTCATCCTTGTAGTCCATACCGGTCTGTGGAGGT-3' |
| pCMV-N-myc-SMARCC2 | Forward | 5’-CCGGAATTCATGGCGGTGCGGAAGAAGGACGGC-3’ |
|  | Reverse | 5’-GCCGCTCGAGTCACTGTGGAGGTGGCACAGGGGT-3’ |
| pCMV-N-myc-SMARCC2-Δ1 | Forward | 5’-CCGGAATTCATCATCATTCCCAGCTACGCTGCC-3’ |
|  | Reverse | 5’-GCCGCTCGAGTCACTGTGGAGGTGGCACAGGGGT-3’ |
| pCMV-N-myc-SMARCC2-Δ2 | Forward | 5’-CCGGAATTCCAGGTGGATGCTGAGAGTCGACCA-3’ |
|  | Reverse | 5’-GCCGCTCGAGTCACTGTGGAGGTGGCACAGGGGT-3’ |
| pCMV-N-myc-SMARCC2-Δ3 | Forward | 5’-CCGGAATTCGACATGCAAAACTTTGGGCTGCGC-3’ |
|  | Reverse | 5’-GCCGCTCGAGTCACTGTGGAGGTGGCACAGGGGT-3’ |
| pCMV-N-myc-SMARCC2-Δ4 | Forward | 5’-CCGGAATTCTACCTGGAGGACTCAGAGGCCTCC-3’ |
|  | Reverse | 5’-GCCGCTCGAGTCACTGTGGAGGTGGCACAGGGGT-3’ |
| pET28A-SMARCC2 | Forward | 5'-CCGGAATTCATGGCGGTGCGGAAGAAGGACGGC-3' |
|  | Reverse | 5'-GCCGCTCGAGTCACTGTGGAGGTGGCACAGGGGT-3' |
| pET28A-SMARCC2-Δ1 | Forward | 5'-CCGGAATTCATCATCATTCCCAGCTACGCTGCC -3' |
|  | Reverse | 5'-GCCGCTCGAGTCACTGTGGAGGTGGCACAGGGGT-3' |
| pET28A-SMARCC2-Δ2 | Forward | 5'-CCGGAATTCCAGGTGGATGCTGAGAGTCGACCA-3' |
|  | Reverse | 5'-GCCGCTCGAGTCACTGTGGAGGTGGCACAGGGGT -3' |
| pET28A-SMARCC2-Δ3 | Forward | 5'-CCGGAATTCGACATGCAAAACTTTGGGCTGCGC-3' |
|  | Reverse | 5'-GCCGCTCGAGTCACTGTGGAGGTGGCACAGGGGT-3' |
| pET28A-SMARCC2-Δ4 | Forward | 5'-CCGGAATTCTACCTGGAGGACTCAGAGGCCTCC-3' |
|  | Reverse | 5'-GCCGCTCGAGTCACTGTGGAGGTGGCACAGGGGT-3' |
| pBiFC-CNBP-VC155 | Forward | 5’-GAAGATCTGGATGAGCAGCAATGAGTGCTTC-3’ |
|  | Reverse | 5’-CCGCTCGAGGGGCTGTAGCCTCAATTGTGCA-3’ |
| pBiFC-SMARCC2-VC155 | Forward | 5’-CCGGAATTCGGATGGCGGTGCGGAAGAAGGAC-3’ |
|  | Reverse | 5’-CCGCTCGAGGCTGTGGAGGTGGCACAGGGGT-3’ |
| pBiFC-SMARCC2-VN173 | Forward | 5’- CCCAAGCTTATGGCGGTGCGGAAGAAGGAC-3’ |
|  | Reverse | 5’-CCGGAATTCGGCTGTGGAGGTGGCACAGGGGT-3’ |
| pBiFC-KPNB1-VN173 | Forward | 5’-ATTTGCGGCCGCGATGGAGCTGATCACCATTCTC-3’ |
|  | Reverse | 5’-TGCTCTAGAAGCTTGGTTCTTCAGTTTCCT-3’ |
| pET28a-CNBP-mCherry | Forward | 5'-TGGACAGCAAATGGGTCGCGGAATGAGCAGCAATGAGTGCTT-3' |
|  | Reverse | 5'-GCCCTTGCTCACCATGGATCCGGCTGTAGCCTCAATTGTGC-3' |
| pET28a-CNBP ΔIDR-mCherry | Forward | 5'-GGATGTTGAAGGAGAATTCGGACACATTCAAAAA-3' |
|  | Reverse | 5'-CGAATTCTCCTTCAACATCCTCCTGAAGATCACA-3' |
| pmCherry-N1-CNBP | Forward | 5'-CCCAAGCTTATGGACATTTGTTATCGCTGTGGTGAG-3' |
|  | Reverse | 5'-CGCGGATCCGCGGCTGTAGCCTCAATTGTGCATTC-3' |
| pmCherry-N1-CNBP ΔIDR | Forward | 5'-GGATGTTGAAGGAGAATTCGGACACATTCAAAAA-3' |
|  | Reverse | 5'-CGAATTCTCCTTCAACATCCTCCTGAAGATCACA-3' |
| pET28a-SMARCC2-EGFP | Forward | 5'-CCGGAATTCATGGCGGTGCGGAAGAAGGA-3' |
|  | Reverse | 5'-CCCAAGCTTCTGTGGAGGTGGCACAGGGG-3' |

CNBP, CCHC-type zinc finger nucleic acid binding protein; KPNB1, karyopherin subunit beta 1; SMARCC2, SWI/SNF related, matrix associated, actin dependent regulator of chromatin subfamily c member 2.

**Table S9 Oligonucleotide sets for short hairpin RNAs and CRISPR-dCas9**

| **Primer set** | **Sequence** |
| --- | --- |
| sh-Scb | 5'-AGGGATACAAGCATATACCACTCGAGTGGTATATGCTTGTATCCCTC-3' (sense); |
|  | 5'-GAGGGATACAAGCATATACCACTCGAGTGGTATATGCTTGTATCCCT-3' (antisense) |
| sh-CNBP #1 | 5’-CCGGTGCACCAAAGTGAAGTGCTATACTCGAGTATAGCACTTCACTTTGGTGCTTTTTG-3’ (sense); |
|  | 5’-GATCCAAAAAGCACCAAAGTGAAGTGCTATACTCGAGTATAGCACTTCACTTTGGTGCA-3’ (antisense) |
| sh-CNBP #2 | 5’-CCGGTCAGCAAGACAAGTGAAGTCAACTCGAGTTGACTTCACTTGTCTTGCTGTTTTTG-3’ (sense); |
|  | 5’-GATCCAAAAACAGCAAGACAAGTGAAGTCAACTCGAGTTGACTTCACTTGTCTTGCTGA-3’ (antisense) |
| sh-CNBP #3 | 5’-CCGGTGCAGATGAGCAGAAATGCTATCTCGAGATAGCATTTCTGCTCATCTGCTTTTTG-3’ (sense); |
|  | 5’-GATCCAAAAAGCAGATGAGCAGAAATGCTATCTCGAGATAGCATTTCTGCTCATCTGCA-3’ (antisense) |
| sh-MYCN #1 | 5’-CCGGGCCAGTATTAGACTGGAAGTTCTCGAGAACTTCCAGTCTAATACTGGCTTTTTG-3’ (sense); |
|  | 5’-AATTCAAAAAGCCAGTATTAGACTGGAAGTTCTCGAGAACTTCCAGTCTAATACTGGC-3’ (antisense) |
| sh-MYCN #2 | 5’-CCGGCAGCAGCAGTTGCTAAAGAAACTCGAGTTTCTTTAGCAACTGCTGCTGTTTTTG-3’ (sense); |
|  | 5’-AATTCAAAAACAGCAGCAGTTGCTAAAGAAACTCGAGTTTCTTTAGCAACTGCTGCTG-3’ (antisense) |
| sh-KPNB1 #1 | 5’-CCGGTCCAGTGTAGTTGTTCGAGATACTCGAGTATCTCGAACAACTACACTGGTTTTTG-3’ (sense); |
|  | 5’-GATCCAAAAACCAGTGTAGTTGTTCGAGATACTCGAGTATCTCGAACAACTACACTGGA-3’ (antisense) |
| sh-KPNB1 #2 | 5’-CCGGTCGGAGATCGAAGACTAACAAACTCGAGTTTGTTAGTCTTCGATCTCCGTTTTTG-3’ (sense); |
|  | 5’-GATCCAAAAACGGAGATCGAAGACTAACAAACTCGAGTTTGTTAGTCTTCGATCTCCGA-3’ (antisense) |
| sh-SMARCC2 #1 | 5’-CCGGTCGCAGTGAAAGCTAAGCACTTCTCGAGAAGTGCTTAGCTTTCACTGCGTTTTTG-3’ (sense); |
|  | 5’-GATCCAAAAACGCAGTGAAAGCTAAGCACTTCTCGAGAAGTGCTTAGCTTTCACTGCGA-3’ (antisense) |
| sh-SMARCC2 #2 | 5’-CCGGTTCACTAAACTGCCGATCAAATCTCGAGATTTGATCGGCAGTTTAGTGATTTTTG-3’ (sense); |
|  | 5’-GATCCAAAAATCACTAAACTGCCGATCAAATCTCGAGATTTGATCGGCAGTTTAGTGAA-3’ (antisense) |
| sh-SMARCC1 #1 | 5’-CCGGTCCCACCACATTTACCCATATTCTCGAGAATATGGGTAAATGTGGTGGGTTTTTG-3’ (sense); |
|  | 5’-GATCCAAAAACCCACCACATTTACCCATATTCTCGAGAATATGGGTAAATGTGGTGGGA-3’ (antisense) |
| sh-SMARCC1 #2 | 5’-CCGGTGCTATGATACTTGGGTCCATACTCGAGTATGGACCCAAGTATCATAGCTTTTTG-3’ (sense); |
|  | 5’-GATCCAAAAAGCTATGATACTTGGGTCCATACTCGAGTATGGACCCAAGTATCATAGCA-3’ (antisense) |
| sh-SMARCA4 #1 | 5’-CCGGTCCATATTTATACAGCAGAGAACTCGAGTTCTCTGCTGTATAAATATGGTTTTTG-3’ (sense); |
|  | 5’-GATCCAAAAACCATATTTATACAGCAGAGAACTCGAGTTCTCTGCTGTATAAATATGGA-3’ (antisense) |
| sh-SMARCA4 #2 | 5’-CCGGTCGGCAGACACTGTGATCATTTCTCGAGAAATGATCACAGTGTCTGCCGTTTTTG-3’ (sense); |
|  | 5’-GATCCAAAAACGGCAGACACTGTGATCATTTCTCGAGAAATGATCACAGTGTCTGCCGA-3’ (antisense) |
| CRISPRa-CNBP #1 | 5’-CACCGCGGCCTTTCACGCCGGGATC-3’ (sense); |
|  | 5’-AAACGATCCCGGCGTGAAAGGCCGC-3’ (antisense) |
| CRISPRa-CNBP #2 | 5’-CACCGGACCCGGATCCCGGCGTGAA-3’ (sense); |
|  | 5’-AAACTTCACGCCGGGATCCGGGTCC-3’ (antisense) |
| CRISPRi-CNBP #1 | 5’-CACCGGCCGTGTGCAGACCCGCGTG-3’ (sense); |
|  | 5’-AAACCACGCGGGTCTGCACACGGCC-3’ (antisense) |
| CRISPRi-CNBP #2 | 5’-CACCGGCGTGTGGCGCAGGCAAGGA-3’ (sense); |
|  | 5’-AAACTCCTTGCCTGCGCCACACGCC-3’ (antisense) |
| CRISPRi-BYSL #1 | 5’-CACCGCGTGCGATCCTTCCCGGCAA-3’ (sense); |
|  | 5’-AAACTTGCCGGGAAGGATCGCACGC-3’ (antisense) |
| CRISPRi-BYSL #2 | 5’-CACCGCCAAATTCAAGGCGGCCCGT-3’ (sense); |
|  | 5’-AAACACGGGCCGCCTTGAATTTGGC-3’ (antisense) |
| CRISPRi-NOP58 #1 | 5’-CACCGGCCTTTTGAGGCCGCGTAGT-3’ (sense); |
|  | 5’-AAACACTACGCGGCCTCAAAAGGCC-3’ (antisense) |
| CRISPRi-NOP58 #2 | 5’-CACCGGTCCTAGTTCCAGTACAGCG-3’ (sense); |
|  | 5’-AAACCGCTGTACTGGAACTAGGACC-3’ (antisense) |
| CRISPRi-RRP9 #1 | 5’-CACCGACAGCGGCTGCTCGTAAGCG-3’ (sense); |
|  | 5’-AAACCGCTTACGAGCAGCCGCTGTC-3’ (antisense) |
| CRISPRi-RRP9 #2 | 5’-CACCGAGAAAGCCGTTCGCTTAACC-3’ (sense); |
|  | 5’-AAACGGTTAAGCGAACGGCTTTCTC-3’ (antisense) |

CNBP, CCHC-type zinc finger nucleic acid binding protein; KPNB1, karyopherin subunit beta 1; SMARCC2, SWI/SNF related matrix associated actin dependent regulator of chromatin subfamily c member 2; SMARCC1, SWI/SNF related matrix associated actin dependent regulator of chromatin subfamily c member 1; SMARCA4, SWI/SNF related matrix associated actin dependent regulator of chromatin subfamily a member 4; BYSL, bystin like; NOP58, NOP58 ribonucleoprotein; RRP9, ribosomal RNA processing 9.
