# Supplementary material for: Therapeutic targeting of CNBP phase separation inhibits ribosome biogenesis and neuroblastoma progression via modulating SWI/SNF complex activity
Source: Clin Transl Med. 2023 Apr 26;13(4):e1235. doi: 10.1002/ctm2.1235 (PMC10131295; doi:10.1002/ctm2.1235)
Supplement: Supplementary file 1 — Supporting Information [file CTM2-13-e1235-s002.doc]

**Supplementary Information**

Therapeutic targeting CNBP phase separation inhibits ribosome biogenesis and neuroblastoma progression via modulating SWI/SNF complex activity

Hu et al.

**Figure S1. Expression profiles of *CNBP* in human tumors.** (**A**) Kaplan-Meier curves indicating the survival of 144 (gencode19, cutoff value=8.82), 102 (GSE3446, cutoff value=9.36), and 88 (GSE16476, cutoff value=10.68) NB patients with high or low *CNBP* expression. (**B**) Mining of a publicly available dataset (ArrayExpress, accession number: E-MTAB-3247) revealing the transcript levels of *Cnbp* in sympathetic ganglia of wild-type (WT, *n*=12) or TH-*MYCN* transgenic mice (*n*=12), and those in TH-*MYCN* transgenic mice of varied ages (*n*=4 for each time point). (**C**) Western blot assay showing the expression of CNBP and MYCN in SH-SY5Y and IMR-32 cells stably transfected with empty vector (mock), *CNBP*, *MYCN*, scramble shRNA (sh-Scb), sh-*CNBP*, or sh-*MYCN*. (**D**) Representative images of immunohistochemical staining indicating the nuclear expression of CNBP (brown, arrowheads) in NB tissues. Scale bars: 100 μm. WD, well differentiated; PD, poorly differentiated. Log-rank test in **A**; unpaired two-sided Student’s *t* test in **B**. Data are shown as representative of three independent experiments in **C** and **D**.

**Figure S2.** ***CNBP* facilitates ribosome biogenesis, protein synthesis, and growth of NB cells.** (**A** and **B**) Western blot assay showing the CNBP protein levels in BE(2)-C, SH-SY5Y and IMR-32 cells stably transfected with scrambe sh-RNA (sh-Scb), sh-*CNBP*, scramble CRISPR (CRISPRa-Scb or CRISPRi-Scb), CRISPRa-*CNBP*, or CRISPRi-*CNBP*. (**C**) Sucrose gradient sedimentation assay indicating the assembly of ribosomal subunits in SH-SY5Y cells stably transfected with empty vector (mock), *CNBP*, sh-Scb, or sh-*CNBP* (*n*=5). (**D** and **E**) AgNOR staining (D) and OP-puro incorporation (E) assays showing the nucleolar NOR dots and protein synthesis of SH-SY5Y cells stably transfected with mock or *CNBP* (*n*=5). Scale bars: 10 μm. (**F** and **G**) Flow cytometry assay indicating apoptosis ratios and cell cycle phases of SH-SY5Y and BE(2)-C cells stably transfected with sh-Scb or sh-*CNBP* (*n*=5). (**H**) Representative images of dissected xenograft tumors formed by subcutaneous injection of SH-SY5Y cells stably transfected with mock, *CNBP*, sh-Scb, or sh-*CNBP* #2 into the dorsal flanks of nude mice (*n*=5 for each group). One-way ANOVA or unpaired two-sided Student’s *t* test in **C-G**. **P<*0.05, ***P<*0.01, ****P<*0.001 vs. mock or sh-Scb. Data are shown as mean ± s.e.m. (error bars) and representative of three independent experiments in **A-G**.

**Figure S3.** **CNBP interacts with KPNB1 or SMARCC2.** (**A**) Coomassie bright blue staining (left panel) and mass spectrometry (right panel) assays revealing the proteins pulled down by CNBP antibody from lysates of IMR-32 cells stably over-expressing *CNBP*. (**B**) Co-IP and western blot assays indicating the interaction of CNBP with KPNB1 or SMARCC2 in IMR-32 cells. The immunoglobulin G (IgG)-bound protein was taken as a negative control. (**C**) Western blot assay showing the expression of KPNB1, SMARCC2, or CNBP in IMR-32 cells stably transfected with empty vector (mock), *KPNB1*, scramble shRNA (sh-Scb), or sh-*KPNB1*. (**D**) Western blot assay indicating the expression of CNBP, KPNB1, or SMARCC2 in IMR-32 cells transfected with scramble CRISPRa (CRISPRa-Scb) or CRISPRa-*CNBP* #1, and those treated with DMSO or IPZ (20 μmol/L) for 24 hours. (**E**) Representative images of soft agar and matrigel invasion assays showing the anchorage-independent growth and invasion capability of SH-SY5Y and IMR-32 cells transfected with scramble CRISPRa (CRISPRa-Scb) or CRISPRa-*CNBP* #1, and those treated with DMSO or IPZ (20 μmol/L) for 24 hours. Data are representative of three independent experiments in **B**-**E**.

**Figure S4.** **CNBP interacts with SWI/SNF subunits via SMARCC2.** (**A**) Schematic diagram showing the assembly of three distinct SWI/SNF complexes, including canonical BRG1/BRM-associated factor (BAF), polybromo-associated BAF (PBAF), and non-canonical BAF (ncBAF). (**B**) Co-IP and western blot assays indicating the interaction between CNBP with SWI/SNF complex subunits SMARCC2, SMARCC1, or SMARCA4 in IMR-32 cells stably transfected with scramble shRNA (sh-Scb) or *sh-CNBP* #2. (**C**) Western blot assay showing the levels of SMARCC2 in SH-SY5Y cells stably transfected with sh-Scb or sh-*SMARCC2*. (**D**) Co-IP and western blot assays indicating the interaction between CNBP with SWI/SNF complex subunits SMARCC2, SMARCC1, or SMARCA4 in SH-SY5Y cells stably transfected with sh-Scb or sh-*SMARCC2*. (**E**) Western blot assay showing the levels of SMARCC1 or SMARCA4 in SH-SY5Y cells stably transfected with sh-Scb, sh-*SMARCC1*, or sh-*SMARCA4*. (**F** and **G**) Schematic illustration (F) and co-IP and western blot assays (G) depicting the domain and interaction between CNBP and SMARCC2 protein in SH-SY5Y cells transfected with Flag-tagged *CNBP* or Myc-tagged *SMARCC2* truncations (upper panel), and that between GST-tagged *CNBP* and His-tagged *SMARCC2* truncation proteins (lower panel). Data are representative of three independent experiments in **B**-**E** and **G**.

**Figure S5.** **CNBP modulates SWI/SNF subunit activity in regulating 18S rRNA processing genes.** (**A**) Schematic depiction (left panel) and Venn diagram (right panel) indicating potential action modes of *CNBP* or *SMARCC2* and their target genes derived from results of RNA-seq and ChIP-seq. (**B**) Heatmap of RNA-seq results (fold change>1.5, *P*<0.05) from IMR-32 cells stably transfected with empty vector (mock) or *CNBP*. (**C**-**E**) ChIP and qPCR (C), dual-luciferase (D), and real-time qRT-PCR (E) assays showing the SMARCC2 enrichment, promoter activity, and transcript levels of target genes *BYSL*, *NOP58*, and *RRP9* in IMR-32 cells stably transfected with mock, *CNBP*, or IDR-deficient *CNBP* (*n*=5). (**F**) By using first antibody specific for SMARCC1, sequential ChIP and qPCR assays indicating relative enrichment of SMARCC1, SMARCA4, or SMARCC2 (normalized to input) on target genes promoters in IMR-32 cells stably transfected with mock, *SMARCC2*, or *CNBP* (*n*=5). (**G** and **H**) Western blot (G) and real-time qRT-PCR (**H**) assays showing the levels of *BYSL*, *NOP58*, and *RRP9* in IMR-32 cells stably transfected with mock, *CNBP*, scramble shRNA (sh-Scb), sh-*SMARCC1*, or sh-*SMARCA4*. (**I**) Kaplan-Meier curves indicating the survival of patients with high or low expression of *SMARCC1* (cutoff values = 9.28 and 6.92) or *SMARCA4* (cutoff values = 9.13 and 7.66) in 249 (TARGET) and 498 (GES62564) NB cases. (**J** and **K**) Western blot assay showing the levels of c-Myc and cyclin D1 (CCND1) in SH-SY5Y cells stably transfected with mock, *CNBP*, *SMARCC2*, sh-Scb, sh-*SMARCC1*, or sh-*SMARCA4*. One-way ANOVA with Bonferroni’s multiple comparison test in **C-F** and **H**. Log-rank test compared the survival difference in **I**. **P<*0.05, ***P<*0.01, ****P<*0.001 vs. mock, mock+mock, or mock+sh-Scb. Data are shown as mean ± s.e.m. (error bars) and representative of three independent experiments in **C-H**, **J** and **K**.

**Figure S6.** ***CNBP* promotes growth and metastasis of NB cells via facilitating target gene expression.** (**A**) Real-time qRT-PCR (normalized to *β-actin*) showing the transcript levels of *BYSL*, *NOP58*, or *RRP9* in SK-SY5Y cells stably transfected with scramble CRISPR (CRISPRa-Scb or CRISPRi-Scb), CRISPRa-*CNBP* #1, CRISPRi-*BYSL*, CRISPRi-*NOP58*, or CRISPRi-*RRP9* (*n*=5). (**B** and **C**) Representative images (B) and quantification (C) of soft agar assay indicating the anchorage-independent growth of SH-SY5Y and IMR-32 cells stably transfected with CRISPRa-Scb, CRISPRi-Scb, CRISPRa-*CNBP* #1, CRISPRi-*BYSL* #1, CRISPRi-*NOP58* #1, or CRISPRi-*RRP9* #1 (*n*=5). (**D** and **E**) Representative images (D) and quantification (E) of matrigel invasion assay indicating the invasion of SH-SY5Y and IMR-32 cells stably transfected with CRISPRa-Scb, CRISPRi-Scb, CRISPRa-*CNBP* #1, CRISPRi-*BYSL* #1, CRISPRi-*NOP58* #1, or CRISPRi-*RRP9* #1 (*n*=5). One-way ANOVA with Bonferroni’s multiple comparison test in **A**, **C** and **E**. ***P<*0.01, ****P<*0.001 vs. a-sgCtrl+i-sgCtrl. ns, non-significant. Data are shown as mean ± s.e.m. (error bars) and representative of three independent experiments in **A**-**E**.

**Figure S7.** **Effects of *CNBP* and macrophages on growth and invasion of NB cells.** (**A**) Quantification of OP-puro incorporation assay indicating nascent protein synthesis of SH-SY5Y cells stably transfected with empty vector (mock), *SMARCC2*, or *CNBP*, and those treated with DMSO or CX-5461 (5 μmol/L) for 48 hours (*n*=5). (**B** and **C**) Representative images (B) and quantification (C) of soft agar and matrigel invasion assays indicating the anchorage-independent growth and invasion of SH-SY5Y and IMR-32 cells stably transfected with mock or *CNBP*, and those treated with DMSO or CX-5461 (5 μmol/L) for 48 hours (*n*=5). (**D**) Immunohistochemical staining revealing F4/80 expression within xenograft tumors in nude mice formed by subcutaneous injection of SH-SY5Y cells stably transfected with mock or *CNBP* (*n* = 5 per group). Scale bars: 100 μm. (**E**) Particle size analysis of extracellular vesicles extracted from SH-SY5Y cells stably transfected with mock, *SMARCC2*, or *CNBP*. (**F**) MTT colorimetric assay depicting the viability of NB cells treated by culture medium (CM) of THP-1 cells or M2 macrophages (*n*=5 per group). (**G** and **H**) Representative images (left panel) and quantification (right panel) of soft agar (G) and matrigel invasion (H) assays showing the anchorage-independent growth and invasion of NB cells treated by CM of THP-1 cells or M2 macrophages (*n* =5 per group). One-way ANOVA or unpaired two-sided Student’s *t* test in **A**, **C**, **D**, **F**, **G** and **H**. ***P<*0.01, ****P<*0.001 vs. mock+mock, mock+DMSO, mock, or THP-1 CM. Data are shown as mean ± s.e.m. (error bars) and representative of three independent experiments in **A**-**C** and **E**-**H**.

**Figure S8.** ***CNBP* promotes tumorigenesis and aggressiveness of NB cells via repressing SMARCC2 activity.** (**A** and **B**) Representative images (A) and immunohistochemical staining (B) showing the size and expression of Ki-67 and CD31 (brown, arrowheads) within xenograft tumors formed by subcutaneous injection of IMR-32 cells stably transfected with empty vector (mock) or *SMARCC2*, and those co-transfected with *CNBP* (*n*=5 for each group). Scale bars: 100 μm. (**C**) Representative images of lung metastatic colonization of nude mice treated with tail vein injection of IMR-32 cells stably transfected with mock or *SMARCC2*, and those co-transfected with *CNBP* (*n* = 5 for each group).

**Figure S9.** **Effects of** i**nhibitory peptide on the viability, ribosome biogenesis, and protein synthesis of NB cells.** (**A**) MTT colorimetric assay showing the viability of NB or MCF 10A cells treated with different doses of CIP-12 or CIP-12 Mut for 48 hours, or those treated with 20 μmol·L^-1^ CIP-12 or CIP-12 Mut for time points as indicated (*n* =5 per group). (**B**) Sucrose gradient sedimentation assay indicating the assembly of ribosomal subunits of BE(2)-C cells treated with CIP-12 or CIP-12 Mut (20 μmol·L^-1^) for 48 hours (*n*=5). (**C**-**F**) AgNOR staining (C), puromycin incorporation (D), OP-puro incorporation (E), soft agar and matrigel invasion (F) assays showing the nucleolar NOR dots, nascent protein synthesis, anchorage-independent growth and invasion capability of of SH-SY5Y and BE(2)-C cells cells treated with CIP-12 or CIP-12 Mut (20 μmol·L^-1^) for 48 hours (*n* = 5). Scale bars, 10 μm. One-way ANOVA with Bonferroni’s multiple comparison test or unpaired two-sided Student’s *t* test in **A**-**C** and **E**. ****P<* 0.001 vs. CIP-12 Mut. ns, non-significant. Data are shown as mean ± s.e.m. (error bars) and representative of three independent experiments in **A**-**F**.

**Figure S10.** **Effects of inhibitory peptide on tumorigenesis and aggressivenesss of NB cells *in vivo*.** (**A**) Representative tumor images, immunohistochemical staining, and AgNOR staining revealing expression of Ki-67, CD31, and F4/80 as well as nucleolar NOR dots (arrowheads) within xenografts formed by subcutaneous injection of BE(2)-C cells in nude mice (*n* = 5 per group) that subsequently treated with intravenous injection of CIP-12 or CIP-12 Mut (3 mg·kg^-1^) as indicated. Scale bars: 100 μm. (**B**) Representative tumor images, hematoxylin & eosin staining (arrowheads), quantification of lung metastatic colonization, and Kaplan-Meier curves of nude mice treated with tail vein injection of BE(2)-C cells and CIP-12 or CIP-12 Mut (3 mg·kg^-1^) as indicated (*n* = 5 for each group). Scale bars: 100 μm. Student’s *t* test in **A** and **B**. Log-rank test for survival comparison in **B**. **P<*0.05, ****P<* 0.001 vs. CIP-12 Mut. Data are shown as mean ± s.e.m. (error bars) in **A** and **B**.

**Figure S11. Kaplan-Meier curves of *BYSL*, *NOP58*, or *RRP9* in NB.** Kaplan-Meier curves indicating the survival of 498 (GSE62564, cutoff values =4.00, 5.49, and 3.41) and 649 (GSE45547, cutoff values = 11.59, 14.33, and 10.33) NB patients with high or low expression of *BYSL*, *NOP58*, or *RRP9*. Log-rank test for survival analysis.

**Figure S12. Raw images of western blot assay in Figure S5J and Figure S5K.**
